# Reviewers in 2024

**DOI:** 10.1098/rspb.2025.0617

**Published:** 2025-05-07

**Authors:** Proceedings B Editorial Team

The Editors of *Proceedings B* thank all reviewers who contributed their time by refereeing manuscripts submitted throughout 2024. From previous feedback, we know that most reviewers value recognition of their efforts by the journal and through services, such as Web of Science Reviewer Recognition Service (previously Publons), which is integrated with *Proceedings B*.

To provide an opportunity for recognition, we list the names of all reviewers (unless they have opted out). This article is made permanent and citable by its digital object identifier (DOI). We encourage you to quote your contribution in your applications for tenure and grants or other forms of research assessment. Where you have provided it, we have included your ORCID, so your contribution can be unambiguously assigned to you.

The team really does appreciate all the work our reviewers do on behalf of the journal, and we look forward to working with you again in 2025.

Aanen D

https://orcid.org/0000-0002-5702-1617

Abbatecola C

Abbott J

https://orcid.org/0000-0002-8743-2089

Abdala V

Abe M

https://orcid.org/0000-0002-0468-1179

Abelson J

Abrahms B

https://orcid.org/0000-0003-1987-5045

Achyuthan H

Ackerley R

https://orcid.org/0000-0003-4621-7929

Acosta DC

Adamo M

https://orcid.org/0000-0001-7571-3505

Adams D

Adelman J

https://orcid.org/0000-0002-5577-8798

Adelson D

https://orcid.org/0000-0003-2404-5636

Adkins-Regan E

Aguirre D

Agüera A

https://orcid.org/0000-0003-0076-1849

Aich U

Akbar S

Akinola M

Albajes R

Albery G

https://orcid.org/0000-0001-6260-2662

Alencar L

Alfani G

Alfaro-Sanchez R

Alizon S

https://orcid.org/0000-0002-0779-9543

Allan B

https://orcid.org/0000-0002-5991-9711

Allegrucci G

Allen B

https://orcid.org/0000-0003-0282-6407

Allen J

Allen W

https://orcid.org/0000-0003-2654-0438

Allritz M

Alma A

https://orcid.org/0000-0002-4908-6533

Almeida SM

Almudí I

Alonso-Alvarez C

https://orcid.org/0000-0002-4765-551X

Alonso-Rodríguez A

Altschul D

Ålund M

https://orcid.org/0000-0003-2861-9721

Álvarez-Presas M

Alves C

https://orcid.org/0000-0003-0501-3532

Amico G

Amoroso C

https://orcid.org/0000-0001-5456-7975

Amos W

https://orcid.org/0000-0002-0971-9914

Anderson L

Anderson R

https://orcid.org/0000-0002-0558-7563

Andreatta D

Andreazzi C

https://orcid.org/0000-0002-9817-0635

Andrew R

Angelini D

https://orcid.org/0000-0002-2776-2158

Angielczyk K

Anthwal N

https://orcid.org/0000-0002-4104-7839

Anton S

Antony B

Apollonio M

Arango A

https://orcid.org/0000-0001-6246-8141

Araya Ajoy Y

Araújo P

https://orcid.org/0000-0003-0435-6218

Arbon J

https://orcid.org/0000-0002-8509-4362

Arce AN

Archie E

https://orcid.org/0000-0002-1187-0998

Arellano S

Arias JS

https://orcid.org/0000-0002-3717-435X

Armbrecht I

Armbruster J

Armbruster S

Armstrong E

Arnold BJ

Arnold D

https://orcid.org/0000-0003-1027-6222

Arnott G

https://orcid.org/0000-0003-1525-3708

Aron S

https://orcid.org/0000-0002-1674-8828

Aronov D

Arostegui M

https://orcid.org/0000-0002-9313-9487

Arranz I

https://orcid.org/0000-0002-1517-1713

Arrenberg A

Asgari D

https://orcid.org/0000-0002-2987-0291

Ash L

https://orcid.org/0000-0002-4242-6494

Ashman T-L

Aslan C

Asnicar D

https://orcid.org/0000-0002-2066-3659

Asplen M

Astegiano J

https://orcid.org/0000-0003-0583-7291

Atmore L

Attwood M

https://orcid.org/0000-0001-5386-1291

Aubier T

https://orcid.org/0000-0001-8543-5596

Audet C

Audet J-N

Audzijonyte A

https://orcid.org/0000-0002-9919-9376

Augustijnen H

Aulsebrook A

https://orcid.org/0000-0002-9217-7043

Aureli F

Avarguès-Weber A

https://orcid.org/0000-0003-3160-6710

Avaria-Llautureo J

https://orcid.org/0000-0002-8610-7428

Aviles J

https://orcid.org/0000-0002-1463-8393

Axelrod C

https://orcid.org/0000-0002-6130-0168

Ayoub N

http://orcid.org/0000-0003-4404-1472

Azar D

Azhar B

Baca M

https://orcid.org/0000-0003-2174-3914

Badde S

https://orcid.org/0000-0002-4005-5503

Baden T

Bagchi B

Bagheri Z

https://orcid.org/0000-0002-1749-3441

Bagley R

Bagnoli F

Bahrndorff S

https://orcid.org/0000-0002-0838-4008

Bai W-N

Baier F

https://orcid.org/0000-0003-4056-1729

Baird E

https://orcid.org/0000-0003-3625-3897

Baker H

Baker J

Bakker T

Balakrishnan R

https://orcid.org/0000-0003-0935-3884

Balas B

Baldan D

Baldassarre D

https://orcid.org/0000-0002-1749-548X

Balfour N

https://orcid.org/0000-0002-2426-3898

Balshine S

Balthasar U

Balthazart J

https://orcid.org/0000-0001-9492-2126

Bamford M

Band N

Banerjee A

Bankston A

Bao M

https://orcid.org/0000-0002-5347-9663

Baran N

https://orcid.org/0000-0001-6270-0625

Barbaro L

https://orcid.org/0000-0001-7454-5765

Barbasch T

https://orcid.org/0000-0002-3439-009X

Barbour A

Barclay P

https://orcid.org/0000-0002-7905-9069

Bard K

Barlow J

Barnard-Kubow KB

Barnett J

https://orcid.org/0000-0001-9789-4132

Barocas A

https://orcid.org/0000-0003-3547-7709

Barott K

https://orcid.org/0000-0001-7371-4870

Barraquand F

https://orcid.org/0000-0002-4759-0269

Barreto E

https://orcid.org/0000-0002-3372-7295

Barrett R

Barrionuevo P

https://orcid.org/0000-0003-1304-8081

Barry M

Barré K

https://orcid.org/0000-0001-5368-4053

Barta Z

Bartley J

Bartol I

Bartomeus I

https://orcid.org/0000-0001-7893-4389

Baskett M

Bastos A

https://orcid.org/0000-0003-3037-687X

Bateman R

Battershill C

Bauch C

https://orcid.org/0000-0001-6214-6601

Baucom R

Bay L

https://orcid.org/0000-0002-9760-2977

Beaman J

https://orcid.org/0000-0002-1618-5308

Beauchamp D

Beaulieu J

Bebbington K

https://orcid.org/0000-0001-7714-9100

Becker D

https://orcid.org/0000-0003-4315-8628

Becker G

Bednarska A

Beechler B

https://orcid.org/0000-0002-9711-4340

Begon M

https://orcid.org/0000-0003-1715-5327

Beijamini F

Bell C

Bell J

https://orcid.org/0000-0001-9996-945X

Bellot S

Bellucci G

Bellvert A

Beltran RS

https://orcid.org/0000-0002-8520-1105

Belyaev R

https://orcid.org/0000-0002-0499-1898

Bemis W

Ben Shaul Y

Benedetti-Cecchi L

Bengtson S

Benichov J

Benoit H

Benowitz K

Benton D

Benton M

https://orcid.org/0000-0002-4323-1824

Berbee M

Berger U

Bergman T

https://orcid.org/0000-0002-9615-5001

Bergmann P

https://orcid.org/0000-0003-4352-9468

Berks H

Berman T

https://orcid.org/0000-0003-4006-794X

Bernal M

Best A

https://orcid.org/0000-0001-6260-6516

Best P

Betran E

https://orcid.org/0000-0002-4742-7963

Bett B

Bettinazzi S

https://orcid.org/0000-0002-1129-0678

Betts M

Beutel R

Bieuville M

Bilde T

Billeter J-C

https://orcid.org/0000-0001-7796-4068

Bindler R

Binning S

https://orcid.org/0000-0002-2804-9979

Biondi E

Biondi L

https://orcid.org/0000-0002-7501-0268

Birk M

Bishop P

https://orcid.org/0000-0003-2702-0557

Bitton P-P

Blackburn G

https://orcid.org/0000-0003-0090-6649

Bladon E

https://orcid.org/0000-0002-0466-0335

Blakeslee A

https://orcid.org/0000-0001-9667-2175

Blanchard G

Blanchet S

https://orcid.org/0000-0002-3843-589X

Blanco F

https://orcid.org/0000-0001-6339-3460

Blanco M

https://orcid.org/0000-0002-8779-1700

Blanco-Sánchez M

Blanke A

https://orcid.org/0000-0003-4385-6039

Blasi D

Blumstein D

https://orcid.org/0000-0001-5793-9244

Boada J

https://orcid.org/0000-0002-3815-625X

Bobrowiec PE

Boco SR

Bodawatta K

https://orcid.org/0000-0002-6095-9059

Bodey T

https://orcid.org/0000-0002-5334-9615

Bogan SN

Boggs C

https://orcid.org/0000-0001-7601-6277

Bogin B

Boisseau R

Boix X

Bok M

https://orcid.org/0000-0002-3169-8523

Bokma F

https://orcid.org/0000-0002-0049-2127

Bond A

Bonisoli Alquati A

https://orcid.org/0000-0002-9255-7556

Bonser S

Boonstra R

https://orcid.org/0000-0003-1959-1077

Booth D

Boots M

https://orcid.org/0000-0003-3763-6136

Boratyński Z

Borg B

Borges R

Born-Torrijos A

https://orcid.org/0000-0002-1258-3616

Bortolus A

Bosco L

Bose C

Boswell KM

Both C

https://orcid.org/0000-0001-7099-9831

Botigue L

Botting J

Boukal D

https://orcid.org/0000-0001-8181-7458

Bourdeau PE

Boutin S

Bowen L

Bowler DE

Bowles AM

https://orcid.org/0000-0002-7487-3811

Boyer C

https://orcid.org/0000-0002-1101-2045

Boyko J

https://orcid.org/0000-0003-0952-169X

Boyles J

https://orcid.org/0000-0003-1494-4515

Bradshaw D

Bradshaw W

Branch T

Brand J

https://orcid.org/0000-0003-3312-941X

Brand P

Brandl H

https://orcid.org/0000-0001-6972-5403

Braun C

Braun E

Brauner C

Bravo F

Brekke P

https://orcid.org/0000-0001-6298-3194

Brenninger F

https://orcid.org/0000-0003-1794-0536

Breton S

https://orcid.org/0000-0002-8286-486X

Brian J

https://orcid.org/0000-0001-9338-4151

Briefer E

https://orcid.org/0000-0003-4147-0319

Briers R

Briffa M

https://orcid.org/0000-0003-2520-0538

Briggs D

https://orcid.org/0000-0003-0649-6417

Briggs S

Brinkman D

Briscoe Runquist R

Brivio F

https://orcid.org/0000-0002-1449-8335

Brochhagen T

Brocklehurst N

https://orcid.org/0000-0003-2650-5480

Brodie III E

Bronstein J

https://orcid.org/0000-0001-9214-1406

Brook C

https://orcid.org/0000-0003-4276-073X

Brooks J

https://orcid.org/0000-0001-6700-8283

Brown G

Brown K

Brown T

Brownstein C

Bruckerhoff L

Brumm H

https://orcid.org/0000-0002-6915-9357

Bruninga-Socolar B

Bruno N

Bruns E

https://orcid.org/0000-0003-4184-0922

Bucciarelli G

Buchsbaum D

https://orcid.org/0000-0002-8716-7756

Buckley C

https://orcid.org/0000-0002-5059-1668

Buckley D

https://orcid.org/0000-0002-6514-2208

Buckling A

Budka M

Buenafe KC

https://orcid.org/0000-0002-1643-5557

Bugnyar T

Bugoni L

Buler J

Burdfield-Steel E

Burdon F

https://orcid.org/0000-0002-5398-4993

Bureš P

Burger J

Burke G

Burke J

Burkett J

https://orcid.org/0000-0002-6357-5499

Burness G

Burns M

Burr D

https://orcid.org/0000-0003-1541-8832

Burt A

https://orcid.org/0000-0001-5146-9640

Burthe S

https://orcid.org/0000-0001-8871-3432

Bush S

Bussière L

Butler G

Butler M

Butts I

Buttstedt A

Buzatto B

https://orcid.org/0000-0002-2711-0336

Byrne M

https://orcid.org/0000-0002-8902-9808

Bythell J

Büring T

https://orcid.org/0000-0003-2557-5217

Büscher T

https://orcid.org/0000-0003-0639-4699

Caccone A

Cadena E

Cai C

https://orcid.org/0000-0002-9283-8323

Cain K

https://orcid.org/0000-0002-6908-7015

Caizergues A

https://orcid.org/0000-0003-4467-3912

Calhim S

https://orcid.org/0000-0001-9059-2641

Callaini G

Callow C

Calvete J

Camacho C

https://orcid.org/0000-0002-9704-5816

Camarero JJ

https://orcid.org/0000-0003-2436-2922

Camerlenghi E

https://orcid.org/0000-0002-6203-069X

Cameron C

https://orcid.org/0000-0002-9810-7476

Cameron H

https://orcid.org/0000-0001-5004-6646

Campbell B

Campbell D

Camus M

https://orcid.org/0000-0003-0626-6865

Cangiano L

Cant J

Cant M

https://orcid.org/0000-0002-1530-3077

Cantalapiedra J

https://orcid.org/0000-0003-0913-7735

cao m

Capellini I

Capilla-Lasheras P

https://orcid.org/0000-0001-6091-7089

Carazo P

https://orcid.org/0000-0002-1525-6522

Cardoso G

https://orcid.org/0000-0001-6258-1881

Carignan C

Carini A

https://orcid.org/0000-0002-0067-6567

Carleton K

https://orcid.org/0000-0001-6306-5643

Carlo T

https://orcid.org/0000-0002-2092-0053

Carlson B

Caron J-B

https://orcid.org/0000-0002-1670-5502

Carretero M

Carroll T

https://orcid.org/0000-0003-0761-1819

Carruthers T

Carter A

https://orcid.org/0000-0001-5550-9312

Carter M

https://orcid.org/0000-0002-5351-8108

Caruso C

Carvalho T

Casacci L

Casagrande S

https://orcid.org/0000-0002-4264-8062

Casas J

https://orcid.org/0000-0003-1666-295X

Cascales-Miñana B

https://orcid.org/0000-0002-3088-835X

Casey C

https://orcid.org/0000-0003-3773-2478

Castellanos MC

https://orcid.org/0000-0002-9967-355X

Castiglione S

Catitti B

https://orcid.org/0000-0003-4018-7300

Cator L

https://orcid.org/0000-0002-6627-1490

Cau A

https://orcid.org/0000-0001-8761-3720

Cavanagh P

Cavigelli S

https://orcid.org/0000-0002-6344-9759

Cease A

https://orcid.org/0000-0002-4778-0775

Cervantes Peralta F

https://orcid.org/0000-0001-7189-4070

Chadwick F

Chakraborty S

Chamberlain D

Chamberlain J

Chan AHH

Chandrasekaran S

https://orcid.org/0000-0002-8405-5708

Chao D-Y

Chaparro-Pedraza C

Chapman M

Charlesworth D

https://orcid.org/0000-0002-3939-9122

Charli J-L

Chase M

Chaston J

Chatar N

https://orcid.org/0000-0003-0449-8574

Chavhan Y

Chen D

Chen K-H

https://orcid.org/0000-0002-8056-5858

Chen P

Chen Z-Q

https://orcid.org/0000-0003-2046-9335

Cheng K

https://orcid.org/0000-0002-4913-2691

Cheng L

https://orcid.org/0000-0002-9974-2973

Cheng W

https://orcid.org/0000-0002-2596-3028

Cheptou P-O

https://orcid.org/0000-0002-5739-5176

Cherry M

Chevin L-M

https://orcid.org/0000-0003-4188-4618

Chiba K

Chikaraishi Y

Childs D

Chippindale A

Chisholm R

https://orcid.org/0000-0002-9847-1710

Chittka L

https://orcid.org/0000-0001-8153-1732

Chouvenc T

https://orcid.org/0000-0003-3154-2489

Chowdhury MAW

https://orcid.org/0000-0003-2893-0026

Christensen-Dalsgaard J

Chu KH

Churro C

Ciccione L

https://orcid.org/0000-0002-5132-2531

Civitello D

https://orcid.org/0000-0001-8394-6288

Claidiere N

https://orcid.org/0000-0002-4472-6597

Clark C

https://orcid.org/0000-0001-7943-9291

Clark H

Clark M

https://orcid.org/0000-0002-3442-3824

Clark R

https://orcid.org/0000-0002-1100-1856

Clayton D

https://orcid.org/0000-0003-1698-3542

Clem CS

Clements C

Clifton I

https://orcid.org/0000-0003-3615-2447

Clinchy M

Clo J

https://orcid.org/0000-0002-3295-9481

Cobain M

Codd J

https://orcid.org/0000-0003-0211-1786

Cohen J

https://orcid.org/0000-0001-9611-9150

Collet J

https://orcid.org/0000-0002-6028-5234

Collins K

https://orcid.org/0000-0002-3379-4201

Collins M

Collins S

https://orcid.org/0000-0003-3856-4285

Colombelli-Negrel D

https://orcid.org/0000-0002-9572-1120

Colombo M

Columbus S

https://orcid.org/0000-0003-1546-955X

Comeault A

Condamine F

https://orcid.org/0000-0003-1673-9910

Condit R

https://orcid.org/0000-0003-4191-1495

Connallon T

https://orcid.org/0000-0002-1962-4951

Connolly S

Connors B

Conradie S

Conway C

Cooney C

https://orcid.org/0000-0002-4872-9146

Cooper L

Cooper N

https://orcid.org/0000-0002-4667-1542

Cordero C

Cordero Rivera A

Cornwall C

Corrado C

Cortesi F

https://orcid.org/0000-0002-7518-6159

Cosme L

Costa F

https://orcid.org/0000-0003-1597-7079

Costa S

Cothran R

Couper L

https://orcid.org/0000-0002-7417-8675

Couzens A

Cox P

Cox R

Cram D

https://orcid.org/0000-0002-8790-8294

Cramer L

Cressler C

https://orcid.org/0000-0002-6281-2798

Crevecoeur F

Cribb A

https://orcid.org/0000-0002-8604-6100

Crofoot M

Crone E

Crone M

Cronin T

https://orcid.org/0000-0001-7375-9382

Cross P

https://orcid.org/0000-0001-8045-5213

Crowley-Gall A

https://orcid.org/0000-0003-2351-7267

Crump A

https://orcid.org/0000-0003-4485-5740

Cruz Laufer A

Csata E

https://orcid.org/0000-0003-2564-9706

Cuenca-Cambronero M

Cueva del Castillo R

https://orcid.org/0000-0002-6385-0729

Cuff J

Cui G

https://orcid.org/0000-0003-4951-1883

Cui R

Culina A

https://orcid.org/0000-0003-2910-8085

Culioli A

https://orcid.org/0009-0004-2253-7228

Cullen T

https://orcid.org/0000-0002-4261-1323

Cummings M

Cunningham C

Currie S

https://orcid.org/0000-0001-5956-0481

Cuthill I

https://orcid.org/0000-0002-5007-8856

Czaczkes TJ

Córdoba Aguilar A

https://orcid.org/0000-0002-5978-1660

D'Aniello B

https://orcid.org/0000-0002-1176-946X

D'Souza D

https://orcid.org/0000-0002-3053-2484

Dadda M

Dahirel M

Dale MRT

Dalerum F

https://orcid.org/0000-0001-9737-8242

Dalesman S

https://orcid.org/0000-0001-8548-3096

Dalle Fratte M

Dang H

Dantzer B

https://orcid.org/0000-0002-3058-265X

Darolti I

Darroch S

https://orcid.org/0000-0003-1922-7136

Datta A

Davenport J

https://orcid.org/0000-0002-9911-2779

Daversa D

https://orcid.org/0000-0002-8984-8897

Davies J

https://orcid.org/0000-0003-3318-5948

Davies N

Davies S

de Angeli Dutra D

https://orcid.org/0000-0003-2341-2035

De Bona S

https://orcid.org/0000-0001-6193-7073

de Laender F

De Lisle S

https://orcid.org/0000-0001-9587-8665

De Monte S

De Petrillo F

De Roode J

De Souza A

https://orcid.org/0000-0002-1124-4874

de Souza Mendonca Jr M

Dean R

Dececchi A

https://orcid.org/0000-0001-7972-3866

Delmore K

https://orcid.org/0000-0003-4108-9729

DeLong J

https://orcid.org/0000-0003-0558-8213

DeLuca W

DeMarche M

Demartsev V

https://orcid.org/0000-0002-1456-9789

Demere T

Denison F

Denoël M

https://orcid.org/0000-0002-3586-8323

Dentzien-Dias P

DeSiervo M

Desurmont G

Devigili A

https://orcid.org/0000-0001-8104-5195

DeVore J

https://orcid.org/0000-0002-6697-9165

Deák B

Diamond K

Dickson B

Dijkerman C

https://orcid.org/0000-0002-5364-3312

Dijkstra P

https://orcid.org/0000-0001-5382-225X

Dillon M

DiMatteo A

Dimitrov D

https://orcid.org/0000-0001-5830-5702

Dingwell J

https://orcid.org/0000-0001-6990-4153

Doekes HM

https://orcid.org/0000-0002-6360-5176

Doherty J-F

Domenech de Cellès M

https://orcid.org/0000-0002-9302-4858

Domning D

Domínguez-Hüttinger E

Donatelli C

Donelan S

https://orcid.org/0000-0002-4066-7884

Donoghue P

Donoso D

Doo S

https://orcid.org/0000-0002-3346-6152

Dorantes-Aranda J

https://orcid.org/0000-0003-1513-6501

Dormann C

Dornhaus A

https://orcid.org/0000-0002-9809-7263

Dossman B

https://orcid.org/0000-0001-9454-1518

Dougherty L

https://orcid.org/0000-0003-1406-0680

Douhard F

https://orcid.org/0000-0002-8503-1089

Dowling A

Downing P

https://orcid.org/0000-0002-5286-3153

Downs C

https://orcid.org/0000-0001-9435-0623

Drake A

Drake J

https://orcid.org/0000-0003-4646-1235

Dreber A

https://orcid.org/0000-0003-3989-9941

Du A

https://orcid.org/0000-0002-7381-5089

Du E

Du Rand E

https://orcid.org/0000-0002-1893-5934

Ducatez S

https://orcid.org/0000-0003-2865-4674

Duchac L

Duchaine B

Duchêne DA

https://orcid.org/0000-0002-5479-1974

Dudley S

https://orcid.org/0000-0002-8660-3720

Duffy K

Duffy M

https://orcid.org/0000-0002-8142-0802

Duguid S

Dujon A

https://orcid.org/0000-0002-1579-9156

Dukas R

https://orcid.org/0000-0003-4533-8542

Duncan C

Duneau D

https://orcid.org/0000-0002-8323-1511

Dunlop R

https://orcid.org/0000-0002-0427-6317

Dunn D

Dunn J

https://orcid.org/0000-0003-3487-6513

Dunne E

https://orcid.org/0000-0002-4989-5904

Dunning L

Dunsworth H

Dupont S

Dupuis J

Durden J

Durgin F

Dusek A

https://orcid.org/0000-0001-8552-4089

Duttke S

Duxbury E

https://orcid.org/0000-0002-5733-3645

Dyble M

Dyer A

https://orcid.org/0000-0002-2632-9061

Dötterl S

https://orcid.org/0000-0001-5228-1332

D’Aloia C

Eastwood J

https://orcid.org/0000-0002-5294-3321

Eck J

Eckert C

Edge M

Edgecombe G

https://orcid.org/0000-0002-9591-8011

Edmunds P

https://orcid.org/0000-0002-9039-9347

Edwards M

Egli L

Eigentler L

https://orcid.org/0000-0002-8333-8132

Eisthen H

Ekhart V

Eldredge N

Elgar M

https://orcid.org/0000-0003-0861-6064

Eliason C

https://orcid.org/0000-0002-8426-0373

Eliassen S

Elliott Smith E

Ellis C

Ellis R

https://orcid.org/0000-0002-3117-0075

Ellis S

https://orcid.org/0000-0001-9019-6040

Ellis Soto D

Ellis V

https://orcid.org/0000-0002-7193-9408

Emberts Z

https://orcid.org/0000-0002-7949-0254

Emery RJN

Emery Thompson M

https://orcid.org/0000-0003-2451-6397

Emlet R

Emmott E

https://orcid.org/0000-0003-4862-179X

Encinas-Viso F

Engel P

Engelen T

Engilis Jr A

Enquist M

https://orcid.org/0000-0003-0198-1288

Esbaugh A

https://orcid.org/0000-0002-7262-4408

Escriva H

Espinosa J

https://orcid.org/0000-0003-0780-2762

Esser H

http://orcid.org/0000-0003-3236-8021

Esteve J

https://orcid.org/0000-0003-2806-2695

Etterson J

Evans J

https://orcid.org/0000-0002-2603-6832

Evans L

Evans S

Ewald P

Fajen B

Falcao R

Fan J

Fargallo JA

Fargevieille A

Farina A

Fariña R

https://orcid.org/0000-0003-0898-0333

Farrell T

https://orcid.org/0000-0002-5005-3536

Fassbinder-Orth C

Faurby S

https://orcid.org/0000-0002-2974-2628

Faust CL

https://orcid.org/0000-0002-8824-7424

Favila ME

Fayet AL

https://orcid.org/0000-0001-6373-0500

Feehan D

Feldblum J

Feng M

https://orcid.org/0000-0001-6772-3017

Fenster C

Fenton A

https://orcid.org/0000-0002-7676-917X

Fenton B

https://orcid.org/0000-0001-9761-5412

Fereres A

Fernandes I

Fernández Domingos E

https://orcid.org/0000-0002-4717-7984

Fernández-Marín H

https://orcid.org/0000-0003-0755-130X

Ferrari M

Ferreira Junior F

https://orcid.org/0000-0002-2034-4121

Feulner P

Feyten L

https://orcid.org/0000-0002-3504-1975

Fichtel C

https://orcid.org/0000-0002-8346-2168

Fidino M

Fiedler K

Field D

https://orcid.org/0000-0002-1786-0352

Field K

https://orcid.org/0000-0002-5196-2360

Figueroa L

https://orcid.org/0000-0003-0655-8278

Fikrig K

https://orcid.org/0000-0002-5850-9948

Firestone C

Fischer E

https://orcid.org/0000-0002-2916-0900

Fiser C

https://orcid.org/0000-0003-1982-8724

Fisher J

Fitzgibbon L

https://orcid.org/0000-0002-8563-391X

Fitzpatrick JL

https://orcid.org/0000-0002-2834-4409

Flack A

https://orcid.org/0000-0002-9099-2802

Fletcher J

Flies A

https://orcid.org/0000-0002-4550-1859

Flintham E

https://orcid.org/0000-0002-8037-5560

Floeter SR

Flores H

Flynn L

https://orcid.org/0000-0001-9795-5521

Foellmer M

https://orcid.org/0000-0003-2331-9754

Foitzik S

https://orcid.org/0000-0001-8161-6306

Fonti P

Foo S

Foo YZ

https://orcid.org/0000-0001-7627-2991

Fooken J

Foote A

https://orcid.org/0000-0001-7384-1634

Foote M

https://orcid.org/0000-0003-3836-5980

Forbes A

https://orcid.org/0000-0001-8332-6652

Ford A

https://orcid.org/0000-0002-4971-048X

Forero-Fuentes D

Forester J

Fornaciai M

https://orcid.org/0000-0003-3350-9439

Fornoff F

Forss S

https://orcid.org/0000-0002-6551-1907

Forster C

Forstmeier W

https://orcid.org/0000-0002-5984-8925

Forsythe A

Fortelius M

Fossion R

https://orcid.org/0000-0001-8456-2075

Foster C

Fowler M

Fowler-Finn K

https://orcid.org/0000-0003-2951-3283

Fox S

https://orcid.org/0000-0002-3207-347X

Fragata I

https://orcid.org/0000-0001-6865-1510

Frago E

Fraimout A

https://orcid.org/0000-0003-4552-3553

Frainer A

https://orcid.org/0000-0002-3703-7152

France L

Francis G

http://orcid.org/0000-0002-8634-794X

Frank H

https://orcid.org/0000-0002-4507-181X

Franklin A

https://orcid.org/0000-0002-8650-8344

Franz M

https://orcid.org/0000-0001-5111-6503

Frean M

Freas C

https://orcid.org/0000-0001-7026-1255

Freitak D

https://orcid.org/0000-0001-8574-0531

Frere C

https://orcid.org/0000-0002-9671-2138

Friberg N

Friberg Nikolai

Frick W

Fricke C

Fricke E

Friedlander T

Friedman S

https://orcid.org/0000-0003-0192-5008

Friedrich M

https://orcid.org/0000-0003-0169-238X

friman v

Fristoe T

Fruciano C

Frye M

https://orcid.org/0000-0003-3277-3094

Fryxell J

https://orcid.org/0000-0002-5278-8747

Fröhlich M

https://orcid.org/0000-0002-1948-7002

Fu F

https://orcid.org/0000-0001-8252-1990

Fuller B

Funamoto D

Funston G

Fusani L

https://orcid.org/0000-0001-8900-796X

Futahashi R

Fuxjager M

https://orcid.org/0000-0003-0591-6854

Gagne R

https://orcid.org/0000-0002-4901-5081

Gaillard J-M

Gaines R

https://orcid.org/0000-0002-3713-5764

Galbraith E

Galic N

Gall G

https://orcid.org/0000-0003-4947-3434

Galtier N

Gamba M

https://orcid.org/0000-0001-9545-2242

Gamboa S

Gammon D

https://orcid.org/0000-0001-6960-111X

Ganel T

Gangloff E

https://orcid.org/0000-0002-9074-8009

Garcia E

Garcia J

https://orcid.org/0000-0002-4183-1404

Garcia Perez C

Garcia-Gonzalez F

https://orcid.org/0000-0001-9515-9038

Garcia-R J

https://orcid.org/0000-0002-6665-9671

García-Rodríguez A

https://orcid.org/0000-0002-9831-2963

Gardner J

Garira W

https://orcid.org/0000-0001-8909-2236

Garnier S

https://orcid.org/0000-0002-3886-3974

Garrett D

Garrido M

https://orcid.org/0000-0001-7899-8913

Garza C

Gatt MC

https://orcid.org/0000-0001-9747-2060

Gatto E

Gaynor K

https://orcid.org/0000-0002-5747-0543

Geary W

Gehman A-L

https://orcid.org/0000-0003-0595-9950

Genner M

https://orcid.org/0000-0003-1117-9168

Ger A

https://orcid.org/0000-0002-6075-5697

Gerardo N

https://orcid.org/0000-0002-3185-6616

Gerritsma Y

https://orcid.org/0000-0002-9002-4000

Gershman S

https://orcid.org/0000-0003-4451-820X

Gettler L

https://orcid.org/0000-0001-7325-8852

Ghaffarzadegan N

Ghedini G

https://orcid.org/0000-0002-5156-2009

Ghezelayagh A

Giachetti C

Giacomelli M

Giaimo S

https://orcid.org/0000-0003-0421-3065

Gibau de Lima a

Gibbon VE

Gibbs J

Gibbs M

Gibert J

https://orcid.org/0000-0002-5083-6418

Gienapp P

Gilby I

Gill R

https://orcid.org/0000-0001-9389-1284

Gillies N

Gillingham MA

Gilman R

https://orcid.org/0000-0003-2000-7966

Gingerich P

https://orcid.org/0000-0002-1550-2674

Girard-Buttoz C

https://orcid.org/0000-0003-1742-4400

Giribet G

https://orcid.org/0000-0002-5467-8429

Giroud S

Giroux A

Giuliani A

https://orcid.org/0000-0002-4640-804X

Gjoni V

https://orcid.org/0000-0003-1740-6093

Gladman N

https://orcid.org/0000-0002-8943-1805

Glastad K

Glazier D

https://orcid.org/0000-0001-7164-1823

Gleichsner A

Gloag R

https://orcid.org/0000-0002-2037-4267

Godbold J

Godde K

https://orcid.org/0000-0001-7888-2945

Godin J-G

https://orcid.org/0000-0001-8465-1721

Godinho RM

https://orcid.org/0000-0003-0107-9577

Godoy P

https://orcid.org/0000-0003-4519-5094

Goedert D

Goettker A

Goldenberg J

Goldey K

Goldstein S

Gomes D

https://orcid.org/0000-0002-2642-3728

Gomez Isaza D

https://orcid.org/0000-0003-3112-8683

Gomez-Mestre I

https://orcid.org/0000-0003-0094-8195

Gomi H

Goncalves A

https://orcid.org/0000-0001-7062-1900

Gonzaga M

Gonzalez A

https://orcid.org/0000-0002-9291-5560

Gonzalez C

Gonzalez-Suarez M

Gonzalez-Voyer A

González-Forero M

https://orcid.org/0000-0003-1015-3089

Goodale E

https://orcid.org/0000-0003-3403-2847

Goodbody-Gringley G

Goold C

Gorczynski D

https://orcid.org/0000-0003-0395-0434

Gordon D

https://orcid.org/0000-0002-1090-9539

Gordon S

Gort G

Gossmann T

Gotanda K

Goto Y

Gotoh H

Goulet T

https://orcid.org/0000-0003-3883-6543

Govaert L

https://orcid.org/0000-0001-8326-3591

Gow E

https://orcid.org/0000-0001-8890-4503

Graham P

Graham S

Graham Z

https://orcid.org/0000-0002-6132-2885

Grainger T

https://orcid.org/0000-0002-6094-0526

Gratton C

Gray A

Gray TNE

Greco I

Green E

https://orcid.org/0009-0006-3168-8452

Greenfield M

https://orcid.org/0000-0003-1935-3423

Greenlee K

Greenway E

Greenwood J

https://orcid.org/0000-0002-6184-0818

Gregorič M

Greig K

Greiner A

https://orcid.org/0000-0002-0471-8031

Grieshop K

https://orcid.org/0000-0001-8925-5066

Griffin A

Griffin J

https://orcid.org/0000-0002-7351-6513

Griffin-Nolan R

https://orcid.org/0000-0002-9411-3588

Griffith O

https://orcid.org/0000-0001-9703-7800

Griffith S

https://orcid.org/0000-0001-7612-4999

Griffiths C

https://orcid.org/0000-0001-7203-0426

Griffiths J

https://orcid.org/0000-0003-0319-515X

Griffiths M

https://orcid.org/0000-0003-4130-9840

Grijseels D

Grime B

Griswold C

https://orcid.org/0000-0003-2993-7043

Groner M

Groot A

https://orcid.org/0000-0001-9595-0161

Gross J

https://orcid.org/0000-0002-0032-1053

Grueter C

https://orcid.org/0000-0001-8770-8148

Grundler M

Grunst A

https://orcid.org/0000-0001-5705-9845

Gruntman M

https://orcid.org/0000-0003-2228-9266

Grüneisen S

Guglielmo C

Guillerme T

Guimaraes PR

Gunderson A

https://orcid.org/0000-0002-0120-4246

Gundiah N

https://orcid.org/0000-0002-9882-5232

Gurka R

https://orcid.org/0000-0002-8907-6663

Guschanski K

Gutiérrez-Cánovas C

Guzik M

Gvozdik L

Gvoždíková Javůrková V

https://orcid.org/0000-0002-9914-0905

Gómez-Bahamón V

Gônet J

Haaland T

Hackett T

Haest B

https://orcid.org/0000-0002-8739-6460

Hage S

https://orcid.org/0000-0002-7018-543X

Hahn A

Haifig I

https://orcid.org/0000-0003-4749-8945

Haig D

Halary S

Hale R

Hall M

https://orcid.org/0000-0002-4738-203X

Hall S

Halliwell C

Halsey L

https://orcid.org/0000-0002-0786-7585

Hamamura T

Hamelin F

Hamilton DG

https://orcid.org/0000-0001-8104-474X

Hamilton M

https://orcid.org/0000-0001-6321-4768

Hampl V

https://orcid.org/0000-0002-5430-7564

Hamylton S

https://orcid.org/0000-0002-6256-3728

Han TA

https://orcid.org/0000-0002-3095-7714

Hanke F

https://orcid.org/0000-0002-1737-3861

Hanlon R

https://orcid.org/0000-0003-0004-5674

hansen B

Hansen MJ

https://orcid.org/0000-0001-7579-9927

Hanson M

https://orcid.org/0000-0002-6125-3672

Harbison S

Harder T

https://orcid.org/0000-0003-2387-378X

Hardy I

https://orcid.org/0000-0002-5846-3150

Harper E

https://orcid.org/0000-0002-1092-3867

Harrington K

Harrington RC

Harris C

https://orcid.org/0000-0003-0977-3774

Harris M

Harris S

Harrison E

https://orcid.org/0000-0002-2050-4631

Harrison H

https://orcid.org/0000-0001-8831-0086

Harrison J

https://orcid.org/0000-0001-5223-216X

Harrison R

Harrison X

https://orcid.org/0000-0002-2004-3601

Harrisson K

Hartfelder K

Hartline D

Hartstone-Rose A

Harvey B

Harvey T

https://orcid.org/0000-0002-2717-7004

Hasenjager M

https://orcid.org/0000-0003-2193-4120

Hasik A

https://orcid.org/0000-0002-4069-7186

Hastings I

Hauert C

https://orcid.org/0000-0002-1239-281X

Haukka A

https://orcid.org/0000-0002-7277-0667

Hausfeld L

https://orcid.org/0000-0002-4396-8201

Haux L

Haven T

Havens H

Hawe R

Hawlena D

Hawlena H

https://orcid.org/0000-0002-0634-2920

Hawley D

Hay M

Hays G

https://orcid.org/0000-0002-3314-8189

Hayward MW

https://orcid.org/0000-0002-5574-1653

Healy K

Heard SB

Heathcote R

Hebets E

https://orcid.org/0000-0002-9382-2040

Hechinger R

https://orcid.org/0000-0002-6633-253X

Heckman R

https://orcid.org/0000-0002-2281-3091

Hector T

Hedelin L

Hedh L

https://orcid.org/0000-0003-0483-3512

Hedrick T

https://orcid.org/0000-0002-6573-9602

Heesen R

https://orcid.org/0000-0002-8730-1660

Hegemann A

https://orcid.org/0000-0002-3309-9866

Heinze S

https://orcid.org/0000-0002-8145-3348

Heiser S

Helantera H

https://orcid.org/0000-0002-6468-5956

Helfrich-Förster C

https://orcid.org/0000-0002-0859-9092

Hellemans S

https://orcid.org/0000-0003-1266-9134

Hellmann J

Helmus M

Hemberger J

Hemmi J

https://orcid.org/0000-0003-4629-9362

Hemmings N

https://orcid.org/0000-0003-2418-3625

Hendrix J

https://orcid.org/0000-0002-8606-1239

Hendry A

Henning J

https://orcid.org/0000-0002-2214-4895

Henrique D

Henshaw J

https://orcid.org/0000-0001-7306-170X

Herborn K

https://orcid.org/0000-0002-5913-7912

Herdegen-Radwan M

https://orcid.org/0000-0001-6593-3256

Herranz M

Herron M

Herzog M

Hessinger DA

Hetem R

https://orcid.org/0000-0003-1953-3520

Heubel K

https://orcid.org/0000-0002-7946-5542

Hex S

https://orcid.org/0000-0002-9142-6917

Hickerson M

Hidalgo M

https://orcid.org/0000-0002-3494-9658

Hiebert Burch S

Higginson A

https://orcid.org/0000-0002-2530-0793

Higham J

https://orcid.org/0000-0002-1133-2030

Hill G

https://orcid.org/0000-0001-8864-6495

Hill J

Hill NJ

Hill R

https://orcid.org/0000-0002-7601-5802

Hillemann F

https://orcid.org/0000-0002-8992-0676

Hindar K

Hindle A

https://orcid.org/0000-0002-1983-9573

Hintz W

https://orcid.org/0000-0002-9755-5314

Hitchcock T

https://orcid.org/0000-0002-6378-5023

Hlusko LJ

Hlásny T

Ho S

Hobson K

Hochmann J-R

Hochscheid S

https://orcid.org/0000-0002-8103-2236

Hoeksema B

Hofmann H

https://orcid.org/0000-0002-3335-330X

Hogle S

Hoh B-P

Holcomb M

Holding M

https://orcid.org/0000-0003-3477-3012

Holl K

Holland L

Holley J-a

Hollis B

Holmes E

https://orcid.org/0000-0001-9596-3552

Holtz T

Holyoak M

Hong R

Hong X-Y

https://orcid.org/0000-0002-5209-3961

Hood W

https://orcid.org/0000-0002-8398-3908

Hooghiemstra H

Hooper P

https://orcid.org/0000-0002-4673-7513

Hoover K

Hope S

https://orcid.org/0000-0002-3711-8593

Hopkins MJ

https://orcid.org/0000-0002-3580-2172

Hopkins S

Hoppitt W

Horváth G

https://orcid.org/0000-0002-0485-333X

Hou C

https://orcid.org/0000-0002-3665-225X

Houslay T

https://orcid.org/0000-0001-5592-9034

Houston DD

Hovel K

How M

https://orcid.org/0000-0001-5135-8828

Howard SR

https://orcid.org/0000-0002-1895-5409

Hočevar S

https://orcid.org/0000-0002-1294-0927

Hrnčíř V

https://orcid.org/0000-0002-6694-8705

Hrushka D

Hsieh C

https://orcid.org/0000-0002-3715-4113

Hsu Y-H

https://orcid.org/0000-0002-0233-5045

Huang H-T

Huang J

https://orcid.org/0000-0003-3247-0253

Huang S

Huckstadt L

https://orcid.org/0000-0002-2453-7350

Hudon J

Hudry K

Hufbauer R

Hughes A

https://orcid.org/0000-0003-2677-1965

Hughey MC

Hull P

https://orcid.org/0000-0001-8607-4817

Hung K-L

https://orcid.org/0000-0002-1557-3958

Hung S-M

https://orcid.org/0000-0002-8908-1497

Hunter M

Huntley J

Hurford A

https://orcid.org/0000-0001-6461-5686

Hurly TA

Huss M

https://orcid.org/0000-0002-5131-6000

Huyseune A

Hyland Bruno J

Häfker NS

Härer A

https://orcid.org/0000-0003-2894-5041

Hébert K

Hörnig MK

Ida T

Iguchi T

Ihara Y

https://orcid.org/0000-0003-3072-2410

Imirzian N

https://orcid.org/0000-0003-4977-0404

Imlay TL

Immonen E

https://orcid.org/0000-0003-1121-6950

Ims R

in 't Zandt D

Ince R

Inoue W

Inouye B

Inouye D

Irvine S

Irwin R

https://orcid.org/0000-0002-1394-4946

Isager P

https://orcid.org/0000-0002-6922-3590

Ishikawa N

https://orcid.org/0000-0002-8268-9512

Isvaran K

Ito K

https://orcid.org/0000-0002-1470-3677

Ivimey-Cook E

https://orcid.org/0000-0003-4910-0443

Iwaniuk A

https://orcid.org/0000-0001-9273-3655

Jaakkola K

https://orcid.org/0000-0002-4113-748X

Jablonski D

Jablonski N

https://orcid.org/0000-0001-7644-874X

Jacobs A

Jacobson P

Jacquin L

https://orcid.org/0000-0002-0521-6113

Jaeggi A

https://orcid.org/0000-0003-1695-0388

Jagiello Z

https://orcid.org/0000-0003-1606-2612

Jain M

https://orcid.org/0000-0002-6258-9629

James L

https://orcid.org/0000-0002-9600-4473

James P

Janes J

Jani A

https://orcid.org/0000-0001-8719-2962

Jansa S

Janssen M

Jaron K

https://orcid.org/0000-0003-1470-5450

Jarzyna M

Jayne B

Jennions M

https://orcid.org/0000-0001-9221-2788

Jensen K

https://orcid.org/0000-0003-0261-3831

Jensen OP

Jerison E

Jeunen G-J

Jewell M

Jheeta S

Jiang L

https://orcid.org/0000-0002-7114-0794

Jimenez A

https://orcid.org/0000-0001-9586-2866

Joanna C

https://orcid.org/0000-0001-7613-8127

Johanson Z

https://orcid.org/0000-0002-8444-6776

Johansson F

https://orcid.org/0000-0002-2302-2603

Johnsen P

Johnson A

https://orcid.org/0000-0002-6447-3179

Johnson D

Johnson P

https://orcid.org/0000-0002-7997-5390

johnston a

https://orcid.org/0000-0001-9026-1199

Jolly C

https://orcid.org/0000-0002-5234-0897

Jones G

Jones T

https://orcid.org/0000-0002-5300-0018

Jonsson B

Jordan C

Jordan N

https://orcid.org/0000-0002-0712-8301

Jordano P

https://orcid.org/0000-0003-2142-9116

Jorgewich-Cohen G

https://orcid.org/0000-0001-9807-6297

Jorquera E

Jorrin B

Jourjine N

https://orcid.org/0000-0002-2537-6093

Joyce W

Jukar A

https://orcid.org/0000-0002-1525-1246

Juma E

Juman M

https://orcid.org/0000-0002-0211-0655

Jung T

Jury C

https://orcid.org/0000-0002-9926-5038

Jusino M

https://orcid.org/0000-0002-3284-4254

Jékely G

https://orcid.org/0000-0001-8496-9836

Jönsson M

Kaandorp J

https://orcid.org/0000-0003-4558-7135

Kalcounis-Rueppell M

Kalinkat G

https://orcid.org/0000-0003-3529-5681

Kalle R

Kallenberg S

Kamilar J

https://orcid.org/0000-0001-6082-9396

Kaminski J

Kano F

https://orcid.org/0000-0003-4534-6630

Kanso E

https://orcid.org/0000-0003-0336-585X

Kanwal J

Kanzaki Y

Kapatsinski V

Kapheim K

https://orcid.org/0000-0002-8140-7712

Karbassioon A

Karjus A

Karsdorp F

https://orcid.org/0000-0002-5958-0551

Kartzinel T

https://orcid.org/0000-0002-8488-0580

Kasada M

https://orcid.org/0000-0001-9023-430X

Kasimatis K

https://orcid.org/0000-0003-0025-5022

Kassian A

Katsuki M

Kauffman M

Kawano S

Kayser M

Kazenel M

https://orcid.org/0000-0002-2101-1062

Keeffe R

Keegan L

Keena M

Keller P

https://orcid.org/0000-0001-7579-6515

Kellermann V

Kelley J

https://orcid.org/0000-0002-8223-7241

Kelley L

https://orcid.org/0000-0003-0700-1471

Kelley N

https://orcid.org/0000-0003-1320-7783

Kelly A

Kelly R

Kenah E

https://orcid.org/0000-0002-7117-7773

Kendal J

https://orcid.org/0000-0001-8644-3117

Kenkel C

Kennedy P

https://orcid.org/0000-0002-2524-6192

Kenrick P

https://orcid.org/0000-0002-3626-5460

Kenyon T

Ketterson E

Khan I

https://orcid.org/0000-0002-0110-2274

Khelifa R

https://orcid.org/0000-0001-6632-8787

Kiat Y

https://orcid.org/0000-0002-3485-3517

Kiel S

https://orcid.org/0000-0001-6281-100X

Kiessling W

https://orcid.org/0000-0002-1088-2014

Kikuchi D

Killam D

https://orcid.org/0000-0001-7569-1828

Kilpatrick AM

https://orcid.org/0000-0002-3612-5775

Kim T

https://orcid.org/0000-0002-9098-073X

Kimmig J

https://orcid.org/0000-0001-8032-4272

Kindsvater H

Kinney M

https://orcid.org/0009-0008-0280-8413

Kirchengast S

Kirchgatter K

Kirchner M

Kirejtshuk AG

Kirkpatrick M

Kitchen A

Kivell T

Klaassen R

http://orcid.org/0000-0002-9840-506X

Klarevas-Irby J

https://orcid.org/0000-0001-7499-4085

Klass K

Klemme I

https://orcid.org/0000-0001-9757-7356

Klepac C

Klug H

https://orcid.org/0000-0003-3169-2452

Kmentova N

Knape J

Knaub J

https://orcid.org/0009-0000-7875-955X

Knell R

Knörnschild M

Koch P

Koch R

https://orcid.org/0000-0002-9068-6231

Kocot K

https://orcid.org/0000-0002-8673-2688

Kofler R

Kohl K

https://orcid.org/0000-0003-1126-2949

Kohli M

Kojima D

Kokko H

https://orcid.org/0000-0002-5772-4881

Kolasa M

kolodny o

https://orcid.org/0000-0002-0095-693X

Konow N

https://orcid.org/0000-0003-3310-9080

Kooyers N

https://orcid.org/0000-0003-3398-7377

Korb J

https://orcid.org/0000-0001-9577-9376

Koren L

https://orcid.org/0000-0002-7425-501X

Korsching S

Kort A

https://orcid.org/0000-0002-2972-2440

Korša A

Koshikawa S

https://orcid.org/0000-0003-2831-555X

Kosmopoulos J

Kotrschal A

https://orcid.org/0000-0003-3473-1402

Koufopanou V

Koul A

https://orcid.org/0000-0002-0917-9583

Koussoroplis A-M

Krach S

Kramer K

https://orcid.org/0000-0002-9157-7758

Krause T

https://orcid.org/0000-0002-8327-3711

Krašovec R

Krebs C

https://orcid.org/0000-0002-2058-2533

Kret M

https://orcid.org/0000-0002-3197-5084

Krishnan A

https://orcid.org/0000-0002-8317-4991

Krishnan N

Kristin T-R

Kruger L

Krupp D

https://orcid.org/0000-0001-6297-7739

Kuemmerli R

Kulahci I

https://orcid.org/0000-0003-0104-0365

Kumata R

https://orcid.org/0000-0002-6428-6357

Kunz J

Kuzawa C

Kyogoku D

https://orcid.org/0000-0002-6214-8241

Labonte D

https://orcid.org/0000-0002-1952-8732

Laborda P

Lacquaniti F

LaDage L

https://orcid.org/0000-0003-1428-1344

Lafferty KD

Laforge M

Laibl L

Laiolo P

https://orcid.org/0000-0002-2009-6797

Lakatos T

Lalor EC

Lambert A

Lameira A

https://orcid.org/0000-0003-3071-4824

Lameris T

Lamichhaney S

LaMontagne J

https://orcid.org/0000-0001-7713-8591

Laméris D

https://orcid.org/0000-0002-3096-7920

Lancia L

Landler L

https://orcid.org/0000-0002-5638-7924

Lane S

https://orcid.org/0000-0002-3797-3178

Langangen Ø

https://orcid.org/0000-0002-6977-6128

Lange E

https://orcid.org/0000-0001-6834-9207

Langley A

Lankinen Å

https://orcid.org/0000-0002-0850-4081

Lappe M

Larsen O

https://orcid.org/0000-0002-8325-0982

Larson J

Lascoux M

Lasley Jr R

https://orcid.org/0000-0001-5607-0938

LaTourrette S

Lau J

Laurin M

Lazzaro B

https://orcid.org/0000-0002-3881-0995

Le Gac M

Leahy L

Ledoux J-B

Lee A

Lee J

Lee J-H

https://orcid.org/0000-0001-9361-1502

Lee M

https://orcid.org/0000-0002-3320-3010

Lee P

Lee W-S

https://orcid.org/0000-0002-2203-6616

Legendre L

https://orcid.org/0000-0003-1343-8725

Lehmann L

https://orcid.org/0000-0001-9549-9577

Lei X

https://orcid.org/0000-0002-3214-3028

Leimar O

https://orcid.org/0000-0001-8621-6977

Lennox R

https://orcid.org/0000-0003-1010-0577

Leonard E

Leonard J

Leone C

Leprieur F

Lerch B

https://orcid.org/0000-0003-4894-6834

Lerman S

Lesku J

https://orcid.org/0000-0001-5073-6954

Leslie A

https://orcid.org/0000-0002-9011-6888

Lester R

https://orcid.org/0000-0003-3566-1266

Leu ST

Leung T

Lewis J

Lewis L

https://orcid.org/0000-0003-1693-5065

Lewis S

Li D

https://orcid.org/0000-0003-0471-7917

Li G

Li J

https://orcid.org/0000-0001-7947-0950

Li Z

Liang W

https://orcid.org/0000-0002-0004-9707

Liao J

https://orcid.org/0000-0002-9520-3235

Libbrecht R

https://orcid.org/0000-0003-4397-000X

Libby E

Lidborg L

Liechti F

https://orcid.org/0000-0001-9473-0837

Lightfoot J

https://orcid.org/0000-0002-5835-2135

Lighton J

Lihoreau M

https://orcid.org/0000-0002-2463-2040

Lima M

https://orcid.org/0000-0002-3700-2945

Limburg K

https://orcid.org/0000-0003-3716-8555

Lin F-C

https://orcid.org/0000-0003-3762-3335

Linck E

Lind P

https://orcid.org/0000-0003-1510-8324

Linden D

Lindner M

Lindstedt C

https://orcid.org/0000-0001-8176-3613

Linnen C

Lisney T

Little C

Liu H

https://orcid.org/0000-0002-8687-3237

Liu J-S

Liu X

https://orcid.org/0000-0002-9168-0659

Liu Y

https://orcid.org/0000-0003-4580-5518

Lo Giudice A

Lo N

Logan M

https://orcid.org/0000-0003-2242-1810

Logue D

https://orcid.org/0000-0003-3020-7101

Lomolino M

Lonero I

Long R

Long T

https://orcid.org/0000-0002-8708-2728

Lopes PC

https://orcid.org/0000-0001-5170-3230

Lopez-Martinez G

https://orcid.org/0000-0002-7937-5002

Lorenzi MC

https://orcid.org/0000-0002-7364-729X

Louvet A

https://orcid.org/0000-0003-1820-5648

Low-Decarie E

Lowi-Merri T

https://orcid.org/0000-0003-3926-5017

Lowndes J

Lowrey B

Loxdale H

Lozano J

Lozier J

Lu C

Lucas E

Lucas J

https://orcid.org/0000-0001-9661-7985

Luchkina E

Lucon-Xiccato T

Lukas D

https://orcid.org/0000-0002-7141-3545

Lukowiak K

Lunau K

https://orcid.org/0000-0001-5184-4201

Lund A

Lundberg M

Lundin O

https://orcid.org/0000-0002-5948-0761

Lunn T

https://orcid.org/0000-0003-4439-2045

Luo Z

Luo Z-X

Lurgi M

https://orcid.org/0000-0001-9891-895X

Luschi P

https://orcid.org/0000-0001-7081-3507

Lučanová M

Lv L

https://orcid.org/0000-0003-2280-1098

Lyamin O

https://orcid.org/0000-0003-2049-0943

Lymbery S

https://orcid.org/0000-0002-8801-7880

Lyn H

https://orcid.org/0000-0002-9746-3030

Lyu S

https://orcid.org/0000-0003-0221-5315

López Romero F

Löfqvist S

Löytynoja A

Ma C-s

https://orcid.org/0000-0002-3253-6921

Maciejewski M

https://orcid.org/0000-0002-2770-787X

Mackay A

https://orcid.org/0000-0002-2924-9657

MacMillan H

https://orcid.org/0000-0001-7598-3273

Macpherson E

Macías García C

https://orcid.org/0000-0003-3242-4214

Madden J

https://orcid.org/0000-0002-0691-0967

Madden M

Madeira D

Maga A

Magalhães S

Magioli M

https://orcid.org/0000-0003-0865-102X

Magle D

Magpantay F

https://orcid.org/0000-0002-1064-0357

Magrath R

https://orcid.org/0000-0002-9109-609X

Magurran A

Maher S

https://orcid.org/0000-0002-3430-0410

Mahoney S

https://orcid.org/0000-0001-8446-423X

Maia A

Maia VC

Maille A

https://orcid.org/0000-0002-5830-3560

Mainwaring MC

Maiorano L

Maisey J

https://orcid.org/0000-0001-7566-0124

Maitner B

Maldonado P

https://orcid.org/0000-0001-9599-1246

Malerbi FK

Malinski K

https://orcid.org/0000-0002-9872-5928

Mallet C

Mamassian P

Manassi M

https://orcid.org/0000-0003-4210-7570

Manenti R

Manikandan B

https://orcid.org/0000-0003-1038-5445

Mannion P

https://orcid.org/0000-0002-9361-6941

Manzini I

https://orcid.org/0000-0002-3575-9637

Marasco V

https://orcid.org/0000-0002-2981-7909

Mariani S

https://orcid.org/0000-0002-5329-0553

Marini G

https://orcid.org/0000-0001-9721-7211

Marini L

Markham C

Marlétaz F

Marquet P

https://orcid.org/0000-0001-6369-9339

Marquitti F

https://orcid.org/0000-0003-0510-3992

Marshall C

https://orcid.org/0000-0001-7832-0950

Marshall D

https://orcid.org/0000-0001-6651-6219

Marshall HH

Martel SI

https://orcid.org/0000-0002-7469-6878

Martin A

Martin D

https://orcid.org/0000-0001-6350-7384

Martin J

https://orcid.org/0000-0001-8704-6076

Martin K

Martin P

https://orcid.org/0000-0003-2611-1211

Martin R

Martin-Creuzburg D

Martin-Ordas G

https://orcid.org/0000-0002-5221-9181

Martinez CM

Martinez de la Puente J

Martinez P

Martino ME

https://orcid.org/0000-0001-5038-5605

Martínez AE

Martínez J

Martínez-Padilla J

Mascaro O

Maslakova S

https://orcid.org/0000-0002-3629-6638

Mason L

https://orcid.org/0000-0001-9567-6262

Mason P

https://orcid.org/0000-0002-5143-666X

Matassa C

https://orcid.org/0000-0003-2632-6191

Matsumiya K

https://orcid.org/0000-0003-3744-6762

Matsuzawa T

https://orcid.org/0000-0002-8147-2725

Matthews P

https://orcid.org/0000-0003-0682-8522

Matz M

May M

May S

https://orcid.org/0000-0002-5312-140X

Mazzucco R

https://orcid.org/0000-0002-7608-0196

McAlister JS

McAvoy A

https://orcid.org/0000-0002-9110-4635

McCallum E

McCann K

McCauley J

McCauley S

https://orcid.org/0000-0001-9649-6693

McCormack J

McCulloch K

https://orcid.org/0000-0002-6203-7381

McCullough E

https://orcid.org/0000-0002-6886-9445

McDonald M

http://orcid.org/0000-0002-5735-960X

McDonough-Goldstein C

https://orcid.org/0000-0001-8949-7479

McElreath MB

McElreath R

McEntee M

https://orcid.org/0000-0002-6616-3596

McGaugh S

https://orcid.org/0000-0003-3163-3436

McGaw I

https://orcid.org/0000-0001-7854-0216

McGechie F

McGetrick J

McGraw EA

https://orcid.org/0000-0001-7973-088X

McGraw P

McGuigan K

https://orcid.org/0000-0002-0525-2202

McGuire L

https://orcid.org/0000-0002-5690-0804

McHenry M

https://orcid.org/0000-0001-5834-674X

McIlroy S

https://orcid.org/0000-0003-2482-8817

McIntyre T

https://orcid.org/0000-0001-8395-7550

McKenzie D

https://orcid.org/0000-0003-0961-9101

McKeon C

McKetchnie A

McKinnon M

Mcknight C

McLain C

McLaren JD

McMahon C

https://orcid.org/0000-0001-5241-8917

McMahon K

McNally L

McNew S

https://orcid.org/0000-0002-1345-1674

McQueen A

https://orcid.org/0000-0002-2375-963X

McRae S

Measey GJ

Medeiros M

https://orcid.org/0000-0002-0744-2018

Medina J

Medley K

Meegaskumbura M

https://orcid.org/0000-0002-4599-6724

Meineke E

Mena S

https://orcid.org/0000-0001-7361-5127

Mendelson T

Mendl M

https://orcid.org/0000-0002-5302-1871

Menezes CT

Menezes J

https://orcid.org/0000-0003-4156-2934

Meng H-H

https://orcid.org/0000-0002-4504-0001

Mensinger A

Merchant H

Merilaita S

https://orcid.org/0000-0001-6437-9863

Merker B

Merks R

https://orcid.org/0000-0002-6152-687X

Merrill TS

Meseguer A

Messeder J

Metcalf C

https://orcid.org/0000-0003-3166-7521

Metz D

https://orcid.org/0000-0002-3502-2416

Metz M

Meunier J

https://orcid.org/0000-0001-6893-2064

Meuti M

https://orcid.org/0000-0003-4607-1392

Meyer JR

https://orcid.org/0000-0001-5566-8452

Meyer M

Michaud M

https://orcid.org/0000-0001-7075-9862

Michel NL

Michod R

https://orcid.org/0000-0002-7782-0379

Michálek O

https://orcid.org/0000-0002-7854-1363

Mihaljevic J

https://orcid.org/0000-0003-2320-5773

Mihlbachler M

Mikkelsen E

Mikula P

https://orcid.org/0000-0002-2731-9105

Milanesi M

https://orcid.org/0000-0001-6244-7455

Miller C

https://orcid.org/0000-0003-1359-5624

Miller E

Miller ET

Miller R

Millien V

https://orcid.org/0000-0002-1390-766X

Millroth P

Milnes M

Milsom W

Milton S

Minias P

https://orcid.org/0000-0002-7742-6750

Mioni G

Miqueleiz I

Mitchell A

Mitchell C

Mitchell E

https://orcid.org/0000-0001-6517-2231

Mitchell K

Mitchell R

Miyashita T

Mizutani C

https://orcid.org/0000-0003-0075-8332

Mladineo I

https://orcid.org/0000-0003-1364-3593

Moe B

https://orcid.org/0000-0002-2306-1899

Moehring A

https://orcid.org/0000-0002-8088-4007

Moeller D

Moleón M

Molleman L

https://orcid.org/0000-0001-7978-7173

Mollon J

https://orcid.org/0000-0001-8533-033X

Momigliano P

https://orcid.org/0000-0001-6390-6094

Mona S

https://orcid.org/0000-0001-9087-0656

Mongeau J-M

https://orcid.org/0000-0002-3292-6911

Mongiardino Koch N

https://orcid.org/0000-0001-6317-5869

Mongle C

https://orcid.org/0000-0002-5474-0501

Montague T

Montazeaud G

https://orcid.org/0000-0002-5249-3404

Montoya P

Mooers A

Moore R

Morales H

https://orcid.org/0000-0002-2964-020X

Moran N

Morea M

Morehouse N

Moreno-Rueda G

https://orcid.org/0000-0002-6726-7215

Moret Y

https://orcid.org/0000-0002-2435-8416

Morimoto J

https://orcid.org/0000-0003-3561-1920

Morimoto T

https://orcid.org/0000-0001-9897-8338

Morin O

https://orcid.org/0000-0002-6216-1307

Morin X

Moritz C

Moroti M

Morris P

Morrison R

https://orcid.org/0000-0001-9161-4734

Morrow EH

Morán-López T

https://orcid.org/0000-0002-5804-5258

Moss J

Mott C

Mottl O

https://orcid.org/0000-0002-9796-5081

Moura M

https://orcid.org/0000-0002-7369-7502

Moura R

https://orcid.org/0000-0002-7911-4734

Moussian B

https://orcid.org/0000-0002-2854-9500

Muir C

https://orcid.org/0000-0003-2555-3878

Mukamel R

Mulhair P

https://orcid.org/0000-0003-3311-4883

Muller L

Muller M

Mulley J

https://orcid.org/0000-0002-1537-7316

Mullon C

https://orcid.org/0000-0002-9875-4227

Munley K

Muntane G

https://orcid.org/0000-0003-1541-8365

Muralidhar P

Murall CL

Muriel J

Murphy K

https://orcid.org/0000-0001-7516-6783

Murray L

https://orcid.org/0000-0002-7810-9546

Murray M

https://orcid.org/0000-0002-2591-0794

Muscarella R

Muth F

https://orcid.org/0000-0003-0904-0589

Mutshinda CM

https://orcid.org/0000-0001-9671-7812

Muzaffar SB

Mylonas D

Mysterud A

https://orcid.org/0000-0001-8993-7382

Mérot C

https://orcid.org/0000-0003-2607-7818

Möller R

Møller A

https://orcid.org/0000-0003-3739-4675

Mühlenhaupt M

https://orcid.org/0000-0003-0234-2533

Naaf T

Nakagawa S

https://orcid.org/0000-0002-7765-5182

Nakawake Y

https://orcid.org/0000-0002-4911-0740

Narayan E

Nardini M

Narwani A

https://orcid.org/0000-0003-4561-0163

Nascimento F

Naug D

https://orcid.org/0000-0001-9252-8037

Nawrot R

https://orcid.org/0000-0002-5774-7311

Nawroth C

Nebauer C

Nedelec S

Neilson R

Nelson K

Nespolo R

https://orcid.org/0000-0001-9337-0606

Ness R

https://orcid.org/0000-0001-7313-8236

Neuenkamp L

Newfield T

Newsome T

https://orcid.org/0000-0003-3457-3256

Newton A

Newton I

Nguyen HN

https://orcid.org/0000-0001-7008-8800

Nicolaï M

https://orcid.org/0000-0002-9570-0311

Niemiller M

Nikola D

Nilsson D-E

https://orcid.org/0000-0003-1028-9314

Niskanen A

https://orcid.org/0000-0003-2017-2718

Nitschke CR

Nittono H

Nityananda V

https://orcid.org/0000-0002-2878-2425

Node-Langlois O

Nolet B

Nonacs P

https://orcid.org/0000-0003-3010-0777

Nooten S

https://orcid.org/0000-0002-1798-315X

Nordahl O

Norderhaug KM

Normark B

Norris D

https://orcid.org/0000-0003-4874-1425

Novoplansky A

Nunn CL

Nunney L

https://orcid.org/0000-0002-4315-3694

Nussey D

https://orcid.org/0000-0002-9985-0317

O'Brien S

O'Connell C

O'Donnell S

https://orcid.org/0000-0002-6338-7148

O'Gorman E

https://orcid.org/0000-0003-4507-5690

O'Hara M

O'Madagain C

https://orcid.org/0000-0002-4086-524X

O'Rourke D

O'Toole A

Oakley C

Oakley CA

Ocampo D

https://orcid.org/0000-0002-7411-2027

Occhibove F

https://orcid.org/0000-0001-7134-8416

Oestreich W

https://orcid.org/0000-0002-0137-5053

Ogihara N

https://orcid.org/0000-0002-1697-9263

Ogino M

https://orcid.org/0000-0002-3780-2942

Ohmer M

Okada Y

Okanishi M

Oldfather M

Oldroyd B

https://orcid.org/0000-0001-6831-3677

Oli M

https://orcid.org/0000-0001-6944-0061

Olito C

https://orcid.org/0000-0001-6883-0367

Oliver J

Oliver K

Oliver R

Ollerton J

Olsson M

https://orcid.org/0000-0002-4130-1323

Olsson S

Ono K

Onstein R

https://orcid.org/0000-0002-2295-3510

Oostra V

Opaev A

Ordin M

https://orcid.org/0000-0002-9464-512X

Organ C

https://orcid.org/0000-0002-2814-2217

Orians C

Orive M

Ørsted M

https://orcid.org/0000-0001-8222-8399

Ortega-Hernández J

https://orcid.org/0000-0002-6801-7373

Ortego J

Ortiz JC

Orton D

Osborn LE

Osiecka AN

https://orcid.org/0000-0001-5392-7895

Osorio DC

Oster H

Osterholtz A

Ostojic L

Ostrander E

Ouyang J

Overcast I

Owens H

https://orcid.org/0000-0003-0071-1745

Paaijmans K

Pahlow M

Painting C

https://orcid.org/0000-0003-0701-2648

Paitz R

https://orcid.org/0000-0003-4609-4359

Palaoro A

https://orcid.org/0000-0002-8629-0728

Pande S

Pantel J

Papastamatiou Y

https://orcid.org/0000-0002-6091-6841

Pappalardo P

Paquette A

Pardi M

https://orcid.org/0000-0001-9766-7511

Paris P

Park J

https://orcid.org/0000-0003-2151-1160

Park T-Y

https://orcid.org/0000-0002-8985-930X

Parker A

Parker B

Parker D

https://orcid.org/0000-0001-7555-5674

Parkos JJ

Parra J

Parry L

Parsch J

Parsons K

https://orcid.org/0000-0003-3355-3587

Parsons M

Parthasarathy R

Patlar B

https://orcid.org/0000-0002-0442-9061

Patterson D

https://orcid.org/0000-0001-7733-4875

Pavlova A

Pawar S

Paz A

Peacor S

https://orcid.org/0000-0002-5334-7775

Pearse I

https://orcid.org/0000-0001-7098-0495

Pearson H

Pedersen A

https://orcid.org/0000-0002-1385-1360

Peecook B

Pegna AJ

Peguero G

https://orcid.org/0000-0002-6464-1486

Pelabon C

https://orcid.org/0000-0002-8630-8983

Pelaez M

Penn D

Pennell M

https://orcid.org/0000-0002-2886-3970

Pennings P

Pennings S

Pennington T

Penton-Voak I

Peoples BK

Peral GT

Pereira Martins L

Perevolotsky T

https://orcid.org/0000-0002-9630-3027

Perez D

https://orcid.org/0000-0002-8560-0368

Perez-Enciso M

http://orcid.org/0000-0003-3524-995X

Perfectti F

https://orcid.org/0000-0002-5551-213X

Peris FP

Perisse E

https://orcid.org/0000-0002-0830-0687

Perkins A

https://orcid.org/0000-0002-7518-4014

Perlman S

Perna A

https://orcid.org/0000-0001-8985-3920

Perrier P

Perry C

https://orcid.org/0000-0001-9398-2418

Perry M

https://orcid.org/0000-0002-5977-8031

Pestana J

Peters S

Petersen J

Petersen R

Petrou E

https://orcid.org/0000-0001-7811-9288

Petsios E

https://orcid.org/0000-0002-7955-7412

Pettersen A

https://orcid.org/0000-0001-6191-6563

Peña J

https://orcid.org/0000-0002-4137-1823

Pfennig K

https://orcid.org/0000-0002-0852-287X

Phillips B

Phillips H

Phillips J

Phillips P

Phillips RD

Philson C

https://orcid.org/0000-0002-5974-347X

Picardi S

https://orcid.org/0000-0002-2623-6623

Pick JL

https://orcid.org/0000-0002-6295-3742

Pietrzak B

https://orcid.org/0000-0001-5446-6277

Pigeon G

https://orcid.org/0000-0002-9166-8633

Pilastro A

https://orcid.org/0000-0001-9803-6308

Pimiento C

Pincheira-Donoso D

Pineda-Einríquez T

Pinheiro F

https://orcid.org/0000-0003-3354-914X

Pintar M

https://orcid.org/0000-0003-0165-3882

Piperno D

Piqueret B

https://orcid.org/0000-0001-6506-8522

Pisor A

https://orcid.org/0000-0001-5780-4542

Pita L

https://orcid.org/0000-0003-0163-1587

Pitnick S

Pizo Ferreira MA

Plotkin J

https://orcid.org/0000-0003-2349-6304

Podos J

https://orcid.org/0000-0002-2546-7999

Poisot T

https://orcid.org/0000-0002-0735-5184

Pokharel S

https://orcid.org/0000-0002-2103-9700

Polly P

Polo-Cavia N

https://orcid.org/0000-0003-4258-0210

Pomiankowski A

https://orcid.org/0000-0002-5171-8755

Pontarp M

https://orcid.org/0000-0001-9045-7829

Ponte G

Pontzer H

https://orcid.org/0000-0003-2397-6543

Popadić A

Porter S

https://orcid.org/0000-0002-4707-9428

Portugal S

https://orcid.org/0000-0002-2438-2352

Post E

https://orcid.org/0000-0002-9471-5351

Postma E

https://orcid.org/0000-0003-0856-1294

Potnis N

Poulin R

https://orcid.org/0000-0003-1390-1206

Powell D

Powell J

Powers M

Powers S

https://orcid.org/0000-0003-0092-808X

Powney G

Prakash A

Pratchett M

https://orcid.org/0000-0002-1862-8459

Pravosudov V

https://orcid.org/0000-0003-1117-7875

Prendergast ME

Pritchard A

https://orcid.org/0000-0001-8722-0011

Proctor D

https://orcid.org/0000-0002-5465-6705

Proffit M

Prugh L

Prétôt L

https://orcid.org/0000-0002-9936-5506

Puckett E

https://orcid.org/0000-0002-9325-4629

Pulido-Silva MT

Purcell J

Purchase C

https://orcid.org/0000-0002-5047-3629

Puschendorf R

Putman N

https://orcid.org/0000-0001-8485-7455

Pyenson N

https://orcid.org/0000-0003-4678-5782

Pyron R

Pérez-Escobar OA

Pérez-Rodríguez A

Püschel T

Qin Y

Queller D

https://orcid.org/0000-0002-5464-1984

Quides K

Quinlan G

https://orcid.org/0000-0002-9814-3936

Quinn J

Quintanilla M

Rabajante J

https://orcid.org/0000-0002-0655-0893

Rabi M

Rabosky D

https://orcid.org/0000-0002-7499-8251

Rader JA

https://orcid.org/0000-0003-2419-3399

Raderschall C

https://orcid.org/0000-0003-2005-1705

Radford C

https://orcid.org/0000-0001-7949-9497

Radwan J

Raffel T

https://orcid.org/0000-0003-2525-0834

Raghavan M

Ragland G

Raia P

https://orcid.org/0000-0002-4593-8006

Railsback S

Raimondi P

Raimondi T

https://orcid.org/0000-0001-6767-1835

Rainey P

https://orcid.org/0000-0003-0879-5795

Rajalakshmi R

Ramesh V

https://orcid.org/0000-0002-0738-8808

Ramirez M

https://orcid.org/0000-0002-9628-8517

Ramm S

https://orcid.org/0000-0001-7786-7364

Ramus F

Rasher D

Rassweiler A

Ratcliff W

Rathore A

https://orcid.org/0000-0002-9585-8274

Rathore S

https://orcid.org/0000-0002-7717-053X

Rattenborg N

https://orcid.org/0000-0002-1140-222X

Ravigne V

Rawlings B

Raymond W

https://orcid.org/0000-0003-3597-2435

Rayner J

https://orcid.org/0000-0001-9259-9046

Rayson H

Reader T

Reale D

https://orcid.org/0000-0002-0419-7125

Recio P

https://orcid.org/0000-0002-5890-0218

Reddin C

https://orcid.org/0000-0001-5930-1164

Reddon A

https://orcid.org/0000-0002-3193-0388

Reddy RB

Reed R

Reed T

Rees B

https://orcid.org/0000-0001-5636-1700

Reid J

https://orcid.org/0000-0002-5007-7343

Reid K

Reilly SB

Reina A

https://orcid.org/0000-0003-4745-992X

Reinhardt J

https://orcid.org/0000-0002-1345-2976

Reis-Santos P

Relyea R

Remacha Sebastian C

https://orcid.org/0000-0002-3763-968X

Remeš V

Renaud L-A

https://orcid.org/0000-0001-8496-3979

Rendell L

https://orcid.org/0000-0002-1121-9142

Renner E

Renner S

https://orcid.org/0000-0003-3704-0703

Resasco J

https://orcid.org/0000-0003-1605-3038

Reschechtko S

Reudink M

Reynolds A

https://orcid.org/0000-0002-7103-3841

Ribak G

https://orcid.org/0000-0002-6267-5471

Ribble D

Rice A

https://orcid.org/0000-0002-8598-9705

Rice P

Richardson D

Richardson J

Richardson M

Ridley A

https://orcid.org/0000-0001-5886-0992

Riebel K

https://orcid.org/0000-0003-2373-8510

Riede F

https://orcid.org/0000-0002-4879-7157

Riede T

https://orcid.org/0000-0001-6875-0017

Riehl C

https://orcid.org/0000-0002-7872-336X

Riesch R

https://orcid.org/0000-0002-0223-1254

Rillo M

https://orcid.org/0000-0002-2471-0002

Rimbach R

https://orcid.org/0000-0003-3059-0382

Ritchie M

Rittschof C

https://orcid.org/0000-0003-0808-0458

Rivers J

https://orcid.org/0000-0001-5041-6002

Rivollat M

Ro T

Roast M

https://orcid.org/0000-0002-9454-7562

Robbins Schug G

Robert T

https://orcid.org/0000-0002-6502-7082

Roberts S

https://orcid.org/0000-0001-5990-9161

Robertson J

Robinson M

https://orcid.org/0000-0001-8025-4024

Robinson P

Robinson S

Roche B

Roche D

Rocke T

Rodd FH

Rodriguez D

Rodriguez Ruano V

Rodriguez Verdugo A

Rodríguez R

https://orcid.org/0000-0003-0262-0839

Rogell B

https://orcid.org/0000-0002-5553-2691

Rogers L

https://orcid.org/0000-0002-9956-1769

Roguz K

Rohilla P

https://orcid.org/0000-0002-1918-9902

Rohner N

Rohner P

https://orcid.org/0000-0002-9840-1050

Rojas B

https://orcid.org/0000-0002-6715-7294

Rokitta S

Roll U

Rollinson N

https://orcid.org/0000-0002-5091-8368

Romano A

https://orcid.org/0000-0002-7502-9268

Romano D

https://orcid.org/0000-0003-4975-3495

Romero-Diaz C

https://orcid.org/0000-0002-0718-4055

Roney J

https://orcid.org/0000-0002-5389-383X

Rong Z

Roopnarine P

https://orcid.org/0000-0002-9811-1176

Roques L

https://orcid.org/0000-0001-6647-7221

Rosa Salva O

Rosas A

Rosas-Luis R

https://orcid.org/0000-0002-7785-7120

Rose G

https://orcid.org/0000-0003-1376-6737

Rosenthal M

https://orcid.org/0000-0003-1291-1728

Ross C

https://orcid.org/0000-0001-7764-761X

Ross P

https://orcid.org/0000-0001-7645-7523

Rossberg A

Rossoni D

Rotjan R

https://orcid.org/0000-0002-3401-9784

Roulston T

Rousselle M

https://orcid.org/0000-0003-3930-0732

Roza A

Rozen D

https://orcid.org/0000-0002-7772-0239

Rubenson J

Rubidge E

https://orcid.org/0000-0001-8997-1177

Rubio De Casas R

https://orcid.org/0000-0003-4276-4968

Rubolini D

Rudman S

Rudstam LG

Ruelens P

https://orcid.org/0000-0003-1524-1897

Runcie D

Rusch H

Russell A

Russo-Tait T

https://orcid.org/0000-0002-2606-2061

Ruszczyk M

Ruta M

Ruthsatz K

https://orcid.org/0000-0002-3273-2826

Rutkowska J

Ruzzante D

https://orcid.org/0000-0002-8536-8335

Råberg L

https://orcid.org/0000-0001-5219-7448

Rössler W

https://orcid.org/0000-0002-5195-8214

Sabat P

Sabiniewicz AL

https://orcid.org/0000-0002-9361-0486

Sackton T

https://orcid.org/0000-0003-1673-9216

Sadd B

https://orcid.org/0000-0003-3136-5144

Sadier A

https://orcid.org/0000-0002-9907-3714

Saenz-Agudelo P

https://orcid.org/0000-0001-8197-2861

Saez A

https://orcid.org/0000-0002-6461-2888

Sakai S

Sakamoto M

Salahshour M

https://orcid.org/0000-0001-5354-9839

Salisbury S

San Mauro D

https://orcid.org/0000-0002-2214-1125

San Millan A

Sanin MJ

Sankey D

Sankey DWE

https://orcid.org/0000-0002-6363-8023

Santoro S

https://orcid.org/0000-0003-0986-3278

Santos E

https://orcid.org/0000-0002-9858-1784

Santos Matos G

Sanz-Aguilar A

Sapir Y

Saranathan V

https://orcid.org/0000-0003-4058-5093

Sarremejane R

Sasa-Marin M

Sasaki T

https://orcid.org/0000-0002-4635-1389

Sato N

https://orcid.org/0000-0001-5023-5375

Sato T

https://orcid.org/0000-0002-4660-9669

Sattherthwaite W

Sauser C

https://orcid.org/0000-0001-8809-781X

Sauve D

https://orcid.org/0000-0002-6318-3719

Scacco M

https://orcid.org/0000-0003-4821-4051

Scaia M

Schank J

https://orcid.org/0000-0002-0811-0103

Scheben A

Schekler I

https://orcid.org/0000-0002-3570-1689

Schenkel M

https://orcid.org/0000-0002-7721-319X

Scheunemann L

Schiebelhut L

https://orcid.org/0000-0002-5417-5426

Schiesari L

Schiffmann C

https://orcid.org/0000-0003-2699-945X

Schilbach L

Schmid D

Schmidt M

Schneemann H

Schneider H

Schneider J

https://orcid.org/0000-0001-8523-7354

Schoeman D

Schoepf V

https://orcid.org/0000-0002-9467-1088

Scholier T

https://orcid.org/0000-0002-0868-1352

Schradin C

https://orcid.org/0000-0002-2706-2960

Schrodt F

https://orcid.org/0000-0001-9053-8872

Schuenemann V

https://orcid.org/0000-0002-8593-3672

Schulte P

https://orcid.org/0000-0002-5237-8836

Schulz N

https://orcid.org/0000-0003-3681-720X

Schwander T

https://orcid.org/0000-0003-1945-5374

Schwanz L

https://orcid.org/0000-0001-5864-7112

Schwartz B

Schweikert L

https://orcid.org/0000-0002-8639-3612

Schwery O

Schäfer R

Schönberg C

Scofield R

https://orcid.org/0000-0002-7510-6980

Scopece G

https://orcid.org/0000-0003-0341-6231

Scribner K

Seamons T

Searle C

https://orcid.org/0000-0002-6607-2299

Seavy N

Secondi J

https://orcid.org/0000-0001-8130-1195

Seebacher F

https://orcid.org/0000-0002-2281-9311

Seebens H

https://orcid.org/0000-0001-8993-6419

Seethapathi N

https://orcid.org/0000-0002-5159-9717

Seger J

Selden P

Selinger J

Serre H

Seymoure B

Sgolastra F

https://orcid.org/0000-0002-2845-8297

Shade A

Shah S

Shahandeh M

Shaner P-J

https://orcid.org/0000-0001-8112-4299

Sharma S

Sheinkopf SJ

Shelomi M

Shen C

Shepard E

https://orcid.org/0000-0001-7325-6398

Shephard A

Shephard S

https://orcid.org/0000-0001-6527-6014

Sheppard P

Sherratt E

https://orcid.org/0000-0003-2164-7877

Sheth S

Shi Z

https://orcid.org/0000-0003-2388-6695

Shikano S

Shimano S

Shiratsuru S

https://orcid.org/0000-0001-8747-9664

Shizuka D

https://orcid.org/0000-0002-0478-6309

Shocket M

https://orcid.org/0000-0002-8995-4446

Shuker D

Sicard M

https://orcid.org/0000-0003-3692-0039

Siebert T

https://orcid.org/0000-0003-4090-5480

Sierro J

https://orcid.org/0000-0003-4628-3117

Sih A

Silk J

Silliman B

Simha A

Simone-Finstrom M

https://orcid.org/0000-0003-2938-9788

Simons A

https://orcid.org/0000-0002-0198-465X

Simova I

Simões P

https://orcid.org/0000-0002-4253-1200

Simões T

singer m

Singh D

https://orcid.org/0000-0002-1202-9826

Singh J

Siopa C

Siqueira A

https://orcid.org/0000-0001-7970-4024

Siracusa ER

https://orcid.org/0000-0003-4205-7278

Siviter H

https://orcid.org/0000-0003-1088-7701

Skeeles M

https://orcid.org/0000-0001-7149-6777

Skinner C

Skirgård H

https://orcid.org/0000-0002-7748-2381

Slater G

https://orcid.org/0000-0002-6306-0911

Smead R

Smeele S

https://orcid.org/0000-0003-1001-6615

Smith H

Smith J

https://orcid.org/0000-0003-4633-4519

Smith K

https://orcid.org/0000-0002-4530-6914

Smith M

https://orcid.org/0000-0001-5660-1727

Smith O

Snekser J

Snyder R

Šobotník J

https://orcid.org/0000-0002-8581-637X

Sokolova I

https://orcid.org/0000-0002-2068-4302

Sol Rueda D

Soldati A

Soltanolkottabi M

Somanathan H

https://orcid.org/0000-0002-4803-3894

Somjee U

https://orcid.org/0000-0002-5130-3605

Sommer-Trembo C

https://orcid.org/0000-0001-9952-2906

Song C

https://orcid.org/0000-0001-7490-8626

Sonne J

https://orcid.org/0000-0002-8570-7288

Soranzo A

Soravia C

Sorte C

Sotelo M

https://orcid.org/0000-0001-9632-9705

Sourav S

https://orcid.org/0000-0002-4172-7711

Sowersby W

https://orcid.org/0000-0002-9774-5935

Spaak J

https://orcid.org/0000-0001-5157-9188

Spady B

http://orcid.org/0000-0002-2426-7805

Speiser D

Spoelstra K

https://orcid.org/0000-0001-8614-4387

Sponsler D

https://orcid.org/0000-0002-4892-9332

Srinivasan M

Srinivasan U

Staab M

https://orcid.org/0000-0003-0894-7576

Stahl A

Stahlschmidt Z

https://orcid.org/0000-0002-6550-4029

Staples T

https://orcid.org/0000-0002-8550-2661

Stapley J

http://orcid.org/0000-0002-9138-5684

Starkloff N

https://orcid.org/0000-0002-0102-8924

Stawski C

https://orcid.org/0000-0003-1714-0301

Stayton T

Stefanescu C

https://orcid.org/0000-0001-8952-7869

Steffan S

https://orcid.org/0000-0002-2219-6409

Steinbrecht RA

Steinmann T

https://orcid.org/0000-0002-2013-7747

Stern J

https://orcid.org/0000-0001-8749-6392

Stevens ED

Stevens P

Stewart A

https://orcid.org/0000-0001-5234-3871

Stewart Merrill T

https://orcid.org/0000-0001-6445-5870

Stewart T

https://orcid.org/0000-0002-7965-5901

Stier A

https://orcid.org/0000-0002-5445-5524

Stigall A

Stiller J

https://orcid.org/0000-0001-6009-9581

Stoffregen T

Stoneking M

Storch D

Storck-Tonon D

Stothart M

https://orcid.org/0000-0002-2863-908X

Stott I

Stowell D

https://orcid.org/0000-0001-8068-3769

Stoy KS

Strait D

https://orcid.org/0000-0002-3572-1663

Strand M

https://orcid.org/0000-0003-1844-7460

Strauss E

https://orcid.org/0000-0003-3413-1642

Strickland K

Strobl M

Strong C

https://orcid.org/0000-0002-6080-9245

Stuhr M

https://orcid.org/0000-0001-9155-9464

Stuligross C

https://orcid.org/0000-0002-5941-0528

Stöger A

Suarez A

https://orcid.org/0000-0002-2257-3366

Suddendorf T

https://orcid.org/0000-0003-3328-7442

Sudyka J

https://orcid.org/0000-0003-3006-3806

Sugiura S

https://orcid.org/0000-0002-9655-2189

Sun B

https://orcid.org/0000-0002-7318-6059

Supple J

Suresh S

Susi H

Sustaita D

https://orcid.org/0000-0001-9932-909X

Suzuki T

Suzuki Y

https://orcid.org/0000-0002-0957-3564

Suárez-Tovar CM

Swaegers J

https://orcid.org/0000-0003-1952-3170

Swarts K

Swedell L

Swedo J

Swiston S

Symons C

https://orcid.org/0000-0003-4120-0327

Szabo B

https://orcid.org/0000-0002-3226-8621

Sztarker J

https://orcid.org/0000-0003-0683-667X

Számadó S

https://orcid.org/0000-0003-2204-9705

Sánchez-Zapata JA

Sørensen M

Taborsky M

https://orcid.org/0000-0002-1357-4316

Taft N

Tahmasian S

Takahashi H

Takahashi T

https://orcid.org/0000-0002-6813-8212

Takeshima Y

Takimoto G

https://orcid.org/0000-0002-4728-4587

Talbot C

https://orcid.org/0000-0003-4604-6891

Tan A

https://orcid.org/0000-0002-9227-4644

Tanentzap A

Tang Q

Tanimoto J

Tapia-Gonzalez JM

Tarasco E

Tarasov S

Tarnita C

Tarwater C

https://orcid.org/0000-0001-7203-1170

Taylor A

Taylor C

http://orcid.org/0000-0003-4299-7104

Taylor R

https://orcid.org/0000-0002-8916-4858

Teears T

Teitelbaum C

https://orcid.org/0000-0001-5646-3184

Temesgen W

ten Cate C

https://orcid.org/0000-0002-4021-8915

Teplitsky C

Terleph T

Terui A

https://orcid.org/0000-0003-3774-2844

Tettamanti G

https://orcid.org/0000-0002-0665-828X

Texeira L

Theobald J

Thessen A

Thia J

https://orcid.org/0000-0001-9084-0959

Thieltges D

Thoma V

https://orcid.org/0000-0002-2688-4639

Thomas A

Thompson M

https://orcid.org/0000-0002-0279-5340

Thompson N

https://orcid.org/0000-0002-9273-3636

Thomson J

Thoral E

https://orcid.org/0000-0001-5218-9907

Thornton A

https://orcid.org/0000-0002-1607-2047

Thuenken T

https://orcid.org/0000-0002-6518-8687

Thygesen U

Tibbetts E

https://orcid.org/0000-0002-5625-892X

Tillmann B

Tilman A

https://orcid.org/0000-0002-6702-7887

Tinius A

Title P

Tkacynski@ljmu.ac.uk P

Tobias J

https://orcid.org/0000-0003-2429-6179

Tobin B

Tobler M

https://orcid.org/0000-0001-5895-6302

Todd B

Todorović D

Tollenaere C

Tomasello M

https://orcid.org/0000-0002-1649-088X

Tomonaga M

https://orcid.org/0000-0002-9319-6991

Tompkins D

Tomášek O

https://orcid.org/0000-0002-2657-5916

Ton R

https://orcid.org/0000-0003-3897-9720

Tonetti V

Tong K

Torices R

Toro-Ibacache V

https://orcid.org/0000-0003-2265-8180

Torres I

Torres L

Torres-Acosta J

Torres-Dowdall J

https://orcid.org/0000-0003-2729-6246

Torres-Martinez L

https://orcid.org/0000-0002-0903-8633

Torson A

Tosi S

https://orcid.org/0000-0001-8193-016X

Toussaint EFA

Trainor L

https://orcid.org/0000-0003-3397-2079

Traniello J

Travis J

Travisano M

https://orcid.org/0000-0001-8168-0842

Treidel L

https://orcid.org/0000-0001-9390-8306

Trigo S

https://orcid.org/0000-0002-9938-6606

Trnka A

Troisi C

Trondrud LM

https://orcid.org/0000-0002-1846-6656

Truskanov N

https://orcid.org/0000-0001-7150-6568

Tryjanowski P

https://orcid.org/0000-0002-8358-0797

Tsang LM

https://orcid.org/0000-0002-5029-5690

Tsessarsky A

Tsuboi M

https://orcid.org/0000-0002-0144-2893

Tuberville T

Tuda M

https://orcid.org/0000-0001-8727-7181

Tuma T

Tungadi T

Tuni C

https://orcid.org/0000-0002-7190-1143

Tunnicliffe V

Turner E

Turner L

Twining JP

Tyukmaeva V

https://orcid.org/0000-0002-1864-5101

Tzortzakaki O

Tütken T

Ujvari B

https://orcid.org/0000-0003-2391-2988

Underwood C

https://orcid.org/0000-0002-5337-3316

Urquhart C

Usherwood J

https://orcid.org/0000-0001-8794-4677

Vale P

https://orcid.org/0000-0003-4558-9202

Valenzuela Sánchez A

Vallejo-Marin M

https://orcid.org/0000-0002-5663-8025

van Beers R

Van Belleghem S

https://orcid.org/0000-0001-9399-1007

van Boekholt B

https://orcid.org/0000-0002-3399-6938

van Breugel F

Van Damme R

https://orcid.org/0000-0002-4585-6940

van de Kamp T

https://orcid.org/0000-0001-7390-1318

van den Berg P

https://orcid.org/0000-0001-7886-9345

van der Bijl W

https://orcid.org/0000-0002-7366-1868

van der Weijden V

https://orcid.org/0000-0002-6413-0045

van Dijk B

Van Dyke J

Van Roy P

van Velzen E

van Vliet S

https://orcid.org/0000-0003-2532-483X

Van Wassenbergh S

https://orcid.org/0000-0001-5746-4621

Vandewege M

https://orcid.org/0000-0003-1115-9087

Vandvik V

https://orcid.org/0000-0003-4651-4798

Vanhov M

Vannette R

https://orcid.org/0000-0002-0447-3468

Vannier J

https://orcid.org/0000-0003-0998-1231

Varela S

Varga T

https://orcid.org/0000-0002-2597-9126

Vargas O

Vasconcelos T

Vasudeva R

https://orcid.org/0000-0002-3831-0384

Vazquez-Salazar A

https://orcid.org/0000-0002-5346-1834

Vedder O

Velez A

Velho T

Veller C

Velloso Missagia R

Velotta J

Verberk W

https://orcid.org/0000-0002-0691-583X

Verde Arregoitia L

https://orcid.org/0000-0001-9520-6543

Veresoglou S

https://orcid.org/0000-0001-6387-4109

Verhulst S

https://orcid.org/0000-0002-1143-6868

Verkerk A

https://orcid.org/0000-0002-3351-8362

Vermeij G

https://orcid.org/0000-0002-6450-5687

Vernier C

https://orcid.org/0000-0002-7381-3833

Viana M

https://orcid.org/0000-0001-5975-6505

Vicovaro M

https://orcid.org/0000-0002-9656-1640

Victor J

Vieira C

https://orcid.org/0000-0001-7433-8360

Vieira-Neto E

Vilcinskas A

https://orcid.org/0000-0001-8276-4968

Vincent J

https://orcid.org/0000-0001-6882-5584

Vinton A

Visser M

https://orcid.org/0000-0002-1456-1939

Vitek N

https://orcid.org/0000-0003-0587-8907

Vogt K

https://orcid.org/0000-0002-8763-342X

Vogwill T

https://orcid.org/0000-0003-1044-6623

Voje K

Volckaert F

Vollstädt M

Volz E

von Schmalensee L

https://orcid.org/0000-0003-3233-4905

Vágási C

https://orcid.org/0000-0002-8736-2391

Vázquez-González C

https://orcid.org/0000-0001-6810-164X

Völter C

Wade A

Wagemans J

Wagner H

https://orcid.org/0000-0002-8191-7595

Wainwright P

Wake M

Walasek N

https://orcid.org/0000-0003-4411-319X

Waldman B

https://orcid.org/0000-0003-0006-5333

Walker R

https://orcid.org/0000-0002-8643-7726

Wall C

Wallace K

Waller D

Waller L

https://orcid.org/0000-0001-5002-8886

Walling C

Walmsley S

https://orcid.org/0000-0001-8577-6884

Walter G

Walter J

https://orcid.org/0000-0003-2983-751X

Wamonje F

Wang B

https://orcid.org/0000-0002-8001-9937

Wang D

https://orcid.org/0000-0002-9045-051X

Wang M

https://orcid.org/0000-0001-8506-1213

Wang Q

Wang R

https://orcid.org/0000-0003-4652-2149

Wang T

wang y

https://orcid.org/0000-0001-8309-5970

Waples R

https://orcid.org/0000-0003-3362-7590

Ware J

https://orcid.org/0000-0002-4066-7681

Warne R

https://orcid.org/0000-0001-7568-074X

Warren E

Wascher C

Watanabe A

https://orcid.org/0000-0001-5057-4772

Watts H

https://orcid.org/0000-0002-9888-825X

Wauters L

Weaver R

https://orcid.org/0000-0002-6160-4735

Weaving H

https://orcid.org/0000-0002-9093-0556

Webb B

https://orcid.org/0000-0002-8336-6926

Webb J

Weber J

Wegmann A

Weinberger V

https://orcid.org/0000-0002-9693-7492

Weinersmith K

https://orcid.org/0000-0002-3886-3480

Weirauch C

http://orcid.org/0000-0003-4492-4515

Weisbecker V

https://orcid.org/0000-0003-2370-4046

Weiss M

Welch K

https://orcid.org/0000-0002-3283-6510

Welicky RL

https://orcid.org/0000-0001-8785-0814

Weller H

Wellman C

https://orcid.org/0000-0001-7511-0464

Wells C

Wendling C

https://orcid.org/0000-0003-4532-7528

Werner A

https://orcid.org/0000-0001-5479-6030

Werner M

Wersebe M

Wesolowski A

https://orcid.org/0000-0001-6320-3575

Wesselingh R

https://orcid.org/0000-0003-0241-2255

Westneat PD

Westra E

https://orcid.org/0000-0003-4396-0354

Westrick S

https://orcid.org/0000-0002-5381-1048

Wetmore L

https://orcid.org/0009-0000-4131-8638

Wetzel WC

Wheatcroft D

Wheeldon T

White A

White S

White T

https://orcid.org/0000-0002-3976-1734

Whiteman N

Whitney D

Wickman J

http://orcid.org/0000-0002-5695-0027

Wielgoss S

https://orcid.org/0000-0002-0127-3380

Wiens J

https://orcid.org/0000-0003-4243-1127

Wikstrom J

Wild G

https://orcid.org/0000-0001-7821-7304

Wild S

Wilkinson C

Wille M

Williams A

Williams H

https://orcid.org/0000-0003-0947-6243

Williams J

Williamson J

https://orcid.org/0000-0003-4916-5386

Willis C

Willmes M

Willmott K

https://orcid.org/0000-0002-9228-0219

Wilson A

Wilson L

https://orcid.org/0000-0001-6659-6001

Wilson M

https://orcid.org/0000-0003-3073-4518

Wilson R

https://orcid.org/0000-0003-3177-0107

Winn A

Wipfler B

https://orcid.org/0000-0002-2367-6789

Wirsing A

https://orcid.org/0000-0001-8326-5394

Wishart A

https://orcid.org/0000-0001-7954-0613

Witek M

Witt C

https://orcid.org/0000-0003-2781-1543

Wittemyer G

https://orcid.org/0000-0003-1640-5355

Woinarski J

Wolff J

https://orcid.org/0000-0003-2326-0326

Wolfner M

Wong M

https://orcid.org/0000-0001-6393-6453

Wood B

https://orcid.org/0000-0002-8187-9429

Woods HA

Worley A

Wright D

https://orcid.org/0000-0002-4066-6718

Wright J

http://orcid.org/0000-0002-5848-4736

Wright MG

Wright S

https://orcid.org/0000-0001-9973-9697

Wu J

https://orcid.org/0000-0002-5784-834X

Wu N

Wuest S

Wuitchik D

https://orcid.org/0000-0002-4090-6400

Wurtsbaugh W

https://orcid.org/0000-0002-1436-5225

Wuster W

Wybouw N

https://orcid.org/0000-0001-7874-9765

Wylie D

Wynn J

https://orcid.org/0000-0002-5552-6435

Wystrach A

Xia C

Xiao S

Xie G

https://orcid.org/0000-0002-7125-7386

Xu Y

https://orcid.org/0000-0003-4420-6353

Xue B

https://orcid.org/0000-0003-0057-0268

Yadav S

Yamamichi M

https://orcid.org/0000-0003-2136-3399

Yamamoto S

https://orcid.org/0000-0002-4162-8457

Yampolsky L

https://orcid.org/0000-0001-7680-6025

Yang L

Yang Q

Yang Z

https://orcid.org/0000-0001-6346-1582

Yannic G

https://orcid.org/0000-0002-6477-2312

Yasuhara M

https://orcid.org/0000-0003-0990-1764

Ye M

Yeh P

Yeung N

Yguel B

Yin D

Yin M

Yohe L

https://orcid.org/0000-0003-1567-8749

Yong J

Yoon HS

https://orcid.org/0000-0001-9507-0105

Yoshioka Y

Young C

https://orcid.org/0000-0001-8919-2093

Young EA

Youngblood M

https://orcid.org/0000-0003-2123-1716

Yousif S

Yovel Y

Yu J-K

https://orcid.org/0000-0001-8591-0529

Yuqui L

Yusuf L

Zaal F

Zabrodskaja A

https://orcid.org/0000-0001-8082-3549

Zaccaro A

Zahnd C

Zaman L

Zanin M

Zayed A

https://orcid.org/0000-0003-3233-4585

Zenil-Ferguson R

Zenit R

https://orcid.org/0000-0002-2717-4954

Zentall T

https://orcid.org/0000-0003-0359-9082

Zhai DY

https://orcid.org/0000-0001-6312-851X

Zhai L

Zhan S

Zhang F

https://orcid.org/0000-0001-6875-654X

Zhang P

Zhang Q-G

https://orcid.org/0000-0001-7128-2664

Zhang S

https://orcid.org/0000-0001-9900-5570

Zhang X

https://orcid.org/0000-0003-1103-2799

Zhang Z

Zhao L

Zhou j

Zhou X

https://orcid.org/0000-0002-2385-8224

Zhou Y

Zhu B

Zhu C

https://orcid.org/0000-0002-0802-7596

Zhulay I

https://orcid.org/0000-0003-4435-4014

Zimmer R

Zimmermann E

Zipple MN

https://orcid.org/0000-0003-3451-2103

Zoidis A

Zöttl M

Zou H-X

https://orcid.org/0000-0002-1965-6910

Zsebok S

Zuo R

https://orcid.org/0000-0002-5582-2306

